# The Cellular and Molecular Basis of Bitter Tastant-Induced Bronchodilation

**DOI:** 10.1371/journal.pbio.1001501

**Published:** 2013-03-05

**Authors:** Cheng-Hai Zhang, Lawrence M. Lifshitz, Karl F. Uy, Mitsuo Ikebe, Kevin E. Fogarty, Ronghua ZhuGe

**Affiliations:** 1 Department of Microbiology and Physiological Systems, University of Massachusetts Medical School, Worcester, Massachusetts, United States of America; 2 Biomedical Imaging Group, University of Massachusetts Medical School, Worcester, Massachusetts, United States of America; 3 Program in Molecular Medicine, University of Massachusetts Medical School, Worcester, Massachusetts, United States of America; 4 Department of Surgery, Division of Thoracic Surgery, University of Massachusetts Memorial Medical Center, Worcester, Massachusetts, United States of America; Monell Chemical Senses Center, United States of America

## Abstract

Bronchodilators are a standard medicine for treating airway obstructive diseases, and β2 adrenergic receptor agonists have been the most commonly used bronchodilators since their discovery. Strikingly, activation of G-protein-coupled bitter taste receptors (TAS2Rs) in airway smooth muscle (ASM) causes a stronger bronchodilation in vitro and in vivo than β2 agonists, implying that new and better bronchodilators could be developed. A critical step towards realizing this potential is to understand the mechanisms underlying this bronchodilation, which remain ill-defined. An influential hypothesis argues that bitter tastants generate localized Ca^2+^ signals, as revealed in cultured ASM cells, to activate large-conductance Ca^2+^-activated K^+^ channels, which in turn hyperpolarize the membrane, leading to relaxation. Here we report that in mouse primary ASM cells bitter tastants neither evoke localized Ca^2+^ events nor alter spontaneous local Ca^2+^ transients. Interestingly, they increase global intracellular [Ca^2+^]_i_, although to a much lower level than bronchoconstrictors. We show that these Ca^2+^ changes in cells at rest are mediated via activation of the canonical bitter taste signaling cascade (i.e., TAS2R-gustducin-phospholipase Cβ [PLCβ]- inositol 1,4,5-triphosphate receptor [IP3R]), and are not sufficient to impact airway contractility. But activation of TAS2Rs fully reverses the increase in [Ca^2+^]_i_ induced by bronchoconstrictors, and this lowering of the [Ca^2+^]_i_ is necessary for bitter tastant-induced ASM cell relaxation. We further show that bitter tastants inhibit L-type voltage-dependent Ca^2+^ channels (VDCCs), resulting in reversal in [Ca^2+^]_i_, and this inhibition can be prevented by pertussis toxin and G-protein βγ subunit inhibitors, but not by the blockers of PLCβ and IP3R. Together, we suggest that TAS2R stimulation activates two opposing Ca^2+^ signaling pathways via Gβγ to increase [Ca^2+^]_i_ at rest while blocking activated L-type VDCCs to induce bronchodilation of contracted ASM. We propose that the large decrease in [Ca^2+^]_i_ caused by effective tastant bronchodilators provides an efficient cell-based screening method for identifying potent dilators from among the many thousands of available bitter tastants.

## Abstract 2

Bitter tastants can activate bitter taste receptors on constricted smooth muscle cells to inhibit L-type calcium channels and induce bronchodilation.

## Author Summary

Bitter taste receptors (TAS2Rs), a G-protein-coupled receptor family long thought to be solely expressed in taste buds on the tongue, have recently been detected in airways. Bitter substances can activate TAS2Rs in airway smooth muscle to cause greater bronchodilation than β2 adrenergic receptor agonists, the most commonly used bronchodilators. However, the mechanisms underlying this bronchodilation remain elusive. Here we show that, in resting primary airway smooth muscle cells, bitter tastants activate a TAS2R-dependent signaling pathway that results in an increase in intracellular calcium levels, albeit to a level much lower than that produced by bronchoconstrictors. In bronchoconstricted cells, however, bitter tastants reverse the bronchoconstrictor-induced increase in calcium levels, which leads to the relaxation of smooth muscle cells. We find that this reversal is due to inhibition of L-type calcium channels. Our results suggest that under normal conditions, bitter tastants can activate TAS2Rs to modestly increase calcium levels, but that when smooth muscle cells are constricted, they can block L-type calcium channels to induce bronchodilation. We postulate that this novel mechanism could operate in other extraoral cells expressing TAS2Rs.

## Introduction

Airway obstructive diseases (asthma and chronic obstructive pulmonary disease [COPD]) have become increasingly prevalent, currently affecting more than 300 million people worldwide. Dysfunction of airway smooth muscle (ASM) cells, a major cell type in the respiratory tree, plays a pivotal role in promoting progression of these diseases and in contributing to their symptoms of these diseases [1]–[3]. With their ability to contract and relax, these cells regulate the diameter and length of conducting airways, controlling dead space and resistance to airflow to and from gas-exchanging areas. Their excessive contraction, as seen in patients with asthma and COPD, can fully close the airways, thereby preventing gas exchange and threatening life. Not surprisingly, bronchodilators have been used as the medication of choice for asthmatic attacks and as a standard medicine for managing COPD [4],[5]. However, available bronchodilators have adverse side effects, and are not sufficiently effective for severe asthmatics and many other COPD patients. A better understanding of the mechanisms regulating ASM thus holds the promise of developing more effective and safe bronchodilators, which in turn would have a significant impact in reducing mortality and morbidity caused by asthma and COPD.

Bitter tastants represent a new class of compounds with potential as potent bronchodilators. Deshpande et al. recently found that cultured ASM cells express G-protein-coupled bitter taste receptors (TAS2Rs) [6], a class of proteins long thought to be expressed only in the specialized epithelial cells in the taste buds of the tongue that allow organisms to avoid harmful toxins and noxious substances characterized by bitterness [7]–[10]. Importantly, bitter tastants with diverse chemical structures cause greater ASM relaxation in vitro than β2 adrenergic agonists, the most commonly used bronchodilators to treat asthma and COPD [6],[11]. Moreover, these compounds can effectively relieve in vivo asthmatic airway obstruction than β2 adrenergic agonists in a mouse model of asthma [6], making them highly attractive bronchodilators for asthma and COPD.

Bitter tastant-induced bronchodilation was unexpected, because these agents appeared to increase intracellular Ca^2+^ concentration ([Ca^2+^]_i_) to a level comparable to that produced by potent bronchoconstrictors [6], which should have led to smooth muscle contraction [12]. To reconcile this apparent paradox, it was proposed that bitter tastants activate the canonical bitter taste signaling pathway (i.e., TAS2R-gustducin-phospholipase Cβ [PLCβ]- inositol 1,4,5-triphosphate receptor [IP3R]) to increase focal Ca^2+^ release from endoplasmic reticulum, which then activate large-conductance Ca^2+^-activated K^+^ channels thereby hyperpolarizing the membrane [6]. However, we subsequently demonstrated through patch-clamp recordings that bitter tastants do not activate large-conductance Ca^2+^-activated K^+^ channels but rather inhibit them [11]. Moreover, three different large-conductance Ca^2+^-activated K^+^ channel blockers did not affect the bronchodilation induced by bitter tastants [11]. Therefore, a different mechanism must be responsible for the bitter tastant-induced bronchodilation.

The apparent conundrum of putative [Ca^2+^]_i_ elevation leading to relaxation may be attributed to the fact that Ca^2+^ responses to bitter tastants were assessed in cultured human ASM cells, while the contractile responses to them were investigated in freshly dissected ASM tissues [6]. It is well known that cultured smooth muscle cell lines alter their phenotype (i.e., losing their ability to contract and relax [13],[14]) and it is likely their Ca^2+^ response is also modified. Therefore, to understand bitter tastant-induced bronchodilation, it is necessary to study the contraction and the underlying signaling in freshly isolated ASM tissues and cells. Using this approach in the present study, we found that bitter tastants activate the canonical bitter taste signaling cascade, slightly increasing global [Ca^2+^]_i_ in resting cells, but not to a level sufficient to cause contraction. However, bitter tastants reverse the increase in [Ca^2+^]_i_ evoked by bronchoconstrictors, leading to bronchodilation. This reversal is mediated by the suppression of L-type voltage-dependent Ca^2+^ channels (VDCCs) in a gustducin βγ subunit-dependent, yet PLCβ- and IP3R-independent manner. Hence, we propose that TAS2R activation in ASM stimulates two opposing Ca^2+^ signaling pathways, both mediated by Gβγ subunits, which increases [Ca^2+^]_i_ at rest but blocks activated L-type VDCCs reversing the contraction they cause. These results provide the cellular and molecular basis of bitter tastant-induced bronchodilation that occurs in vitro and in vivo. They further reveal a Ca^2+^ signal that is well suited for screening and identifying potent bronchodilators from among the many thousands of available bitter tastants.

## Results

To uncover the mechanism underlying bitter tastant-induced bronchodilation as demonstrated in both in vitro and in vivo normal and asthmatic models of mice, and in vitro human airways ([6],[11],[15],[16], we examined how bitter tastants affected both [Ca^2+^]_i_ and ASM contraction in freshly isolated airway cells and tissues from mouse and human. Fluo-3 was used to assess the effect of bitter tastants on [Ca^2+^]_i_; chloroquine and denatonium, two substances commonly used to study bitter taste signaling, were used as bitter tastants.

### Bitter Tastants Modestly Raise Global [Ca^2+^]_i_ with No Change in Force Generation in Native ASM at Rest

**Figure 1:**
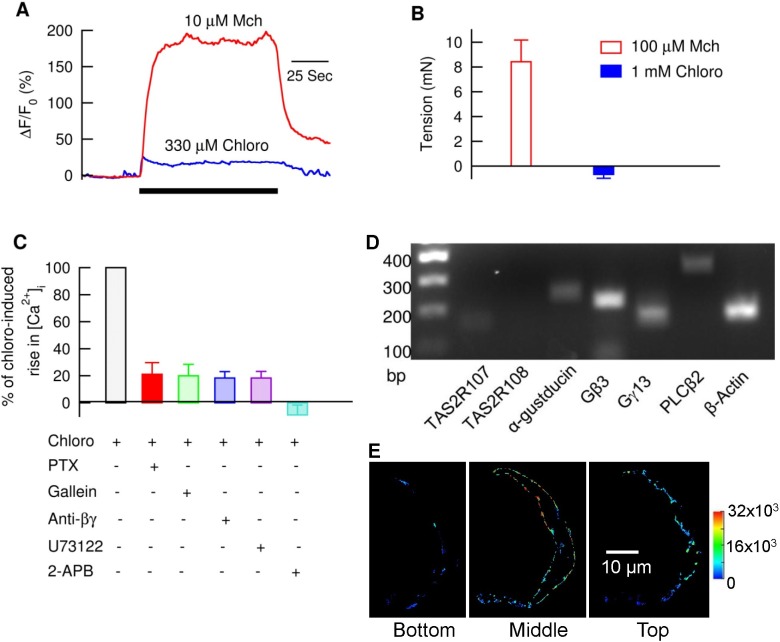
Bitter tastants modestly increase intracellular Ca^2+^ concentration ([Ca^2+^]_i_) by activating a canonical TAS2R signaling cascade. (A) Chloroquine (Chloro) raised [Ca^2+^]_i_ to a level much less than Mch. [Ca^2+^]_i_ was measured with fluo-3 in the form of acetoxymethyl ester, loaded into isolated mouse ASM cells, and expressed as ΔF/F_0_ (%). (B) 1 mM chloro did not contract airways (using tension as its proxy) while 100 µM Mch caused a robust contraction. Data are mean ± SEM (*n* = 6 for chloro, and *n* = 5 for Mch). (C) PTX, gallein, anti-βγ (MPS-phosducin-like protein C terminus, a Gβγ blocking peptide), U73122, and 2-APB inhibited chloro-induced increase in [Ca^2+^]_i_ (*n* = 19–24 cells). Isolated mouse ASM cells were either pretreated with 1 µg/ml PTX for 6–8 h or with 1 µM anti-βγ for 1–2 h or with each of the other compounds listed for 5–10 min. The effects of PTX and anti-βγ were calculated by normalizing the response of chloro to that from the time matched cells without the pretreatments, and the effects of other three compounds were analyzed by normalizing the response of chloro to its own control without the compound. (D) RT-PCR transcripts after amplification with primers to TAS2R107, α-gustducin, Gβ3, Gγ13, PLCβ2, and β-actin. Note that no transcript was detected with TAS2R108 primers. RNAs were isolated from mouse tracheas and mainstem bronchi, and reactions without complementary DNA were used as a negative control. (E) Cellular distribution of TAS2R107 in three focus planes (bottom, middle, and top) of an isolated mouse ASM cell. The TAS2R107 immunostaining intensity after 3D deconvolution (see Methods) was pseudocolored with the color map on the right. This makes positive (but dim) pixels more easily distinguished from background. Eight cells showed a similar subcellular distribution pattern.

We started our analysis by examining the Ca^2+^ response to bitter tastants in resting cells. In contrast to the marked increase in global [Ca^2+^]_i_ reported in resting cultured human ASM cells [6], we observed, in resting native ASM cells from mouse, that chloroquine (0.1 µM–1 mM) only modestly raised global [Ca^2+^]_i_ (and to a level much lower than when cells contracted after application of Mch at 0.1 µM–100 µM) (Figure 1A and Figure S1A). Chloroquine (330 µM) increased fluo-3 fluorescence (ΔF/F_0_) (i.e., [Ca^2+^]_i_) both in the presence of extracellular Ca^2+^ (37.7%±8%, *n* = 19) and in its absence (29.3%±6%, *n* = 15; *p*>0.05), indicating that the source for this chloroquine response is from internal Ca^2+^ stores.

To examine whether this modest increase in [Ca^2+^]_i_ is sufficient to trigger contraction, we measured smooth muscle force formation in mouse airways. As shown in (Figure 1B and S1B), chloroquine (10 µM–1 mM) did not cause contraction of mouse airways, although there was a tendency to decrease the basal tone of airways. As a comparison, Mch at concentrations between 0.3 µM and 10 µM induced contraction markedly and in a dose-dependent manner (Figure 1B and S1B).

### Bitter Tastants Do Not Generate Localized Ca^2+^ Events

Mouse ASM cells exhibit spontaneous Ca^2+^ sparks resulting from the opening of ryanodine receptors in the sarcoplasmic reticulum [17]. To test whether bitter tastants generate local Ca^2+^ events as proposed by Deshpande et al. [6], we stimulated ASM cells with chloroquine (10 µM, a concentration around EC50) for 2 min and measured Ca^2+^ sparks. Off 40 chloroquine-stimulated cells, 27 cells generated a global [Ca^2+^]_i_ increase that precluded an accurate estimate of Ca^2+^ sparks. In the remaining 13 cells without a detectable global rise in [Ca^2+^]_i_, chloroquine inhibited the spark frequency but had no effect on the amplitude (frequency [Hz]: 2.13±0.24 in control and 1.62±0.21 with chloroquine [*n* = 13, *p*<0.05, paired Student's *t*-test]; amplitude [ΔF/F_0_ at the brightest location]: 20.6±1.69 in control and 18.1±1.3 with chloroquine [*n* = 13, *p*>0.05, paired Student's *t*-test]). To test whether spontaneous Ca^2+^ sparks mask the effect of bitter tastants on other forms of local Ca^2+^ releases, such as Ca^2+^ puffs due to the opening of IP3Rs [18], we examined the Ca^2+^ responses to chloroquine in ASM cells pretreated with 100 µM ryanodine. In these cells, prior to chloroquine application, no spontaneous sparks were observed (*n* = 14). Chloroquine (10 µM) increased global [Ca^2+^] by 12%±4% (ΔF/F_0_ at its brightest location) in nine cells, and failed to cause any detectable Ca^2+^ increase in five cells. There were no detectable local Ca^2+^ events produced in any of the 14 cells. These results indicate that chloroquine at 10 µM does not increase local Ca^2+^ events (either Ca^2+^ puffs or Ca^2+^ sparks).

### Bitter Tastants Activate the TAS2R Signaling Pathway to Modestly Raise Global [Ca^2+^]_i_ in Native ASM at Rest

We next examined the cause of the modest global [Ca^2+^]_i_ rise by bitter tastants. Since in taste cells, bitter tastants bind to TAS2R to activate the pertussis toxin (PTX) sensitive G-protein gustducin, which in turn induces a PLCβ2 and IP3 signaling cascade [19],[20], we studied whether bitter tastants activate this TAS2R signaling pathway. In native ASM cells, PTX (1 µg/ml, and 6–8 h pretreatment), reduced the chloroquine-induced increase in global [Ca^2+^]_i_ to 21.1%±8.6% of the control cells (*n* = 20; Figure 1C). Also both gallein (20 µM and 30 min pretreatment), a blocker of the Gβγ dimer of PTX sensitive G proteins, and MPS-phosducin-like protein C terminus, a Gβγ blocking peptide (anti-βγ; 1 µM, and 1 h pretreatment) [21],[22] reduced the bitter tastant-mediated increase in [Ca^2+^]_i_ to 19.9%±8.5% (*n* = 19; Figure 1C) and 18.4%±4.8% of the controls, respectively. Finally, U73122 (3 µM), a blocker of PLCβ, and 2-aminoethoxydiphenyl borate (2-APB) (50 µM), an IP3R antagonist, suppressed the bitter tastant-induced increases in [Ca^2+^]_i_ to 18.0%±5.5% (*n* = 24) and −10.5%±7.3% of controls, respectively (Figure 1C). These results indicate that bitter tastants do activate the TAS2R signaling transduction pathway (i.e., TAS2R-PTX-sensitive G protein-PLCβ-IP3R) to release Ca^2+^ from internal stores. This conclusion is further supported by the finding that mouse ASM cells express transcripts for TAS2R107, α-gustducin, Gβ3, Gγ13, and PLCβ2 (Figure 1D), and display peripheral localization of TAS2R107 (Figure 1E).

### Bitter Tastant-Induced Bronchodilation Is Due to Reversal of the Rise in Global [Ca^2+^]_i_ Caused by Bronchoconstrictors

**Figure 2:**
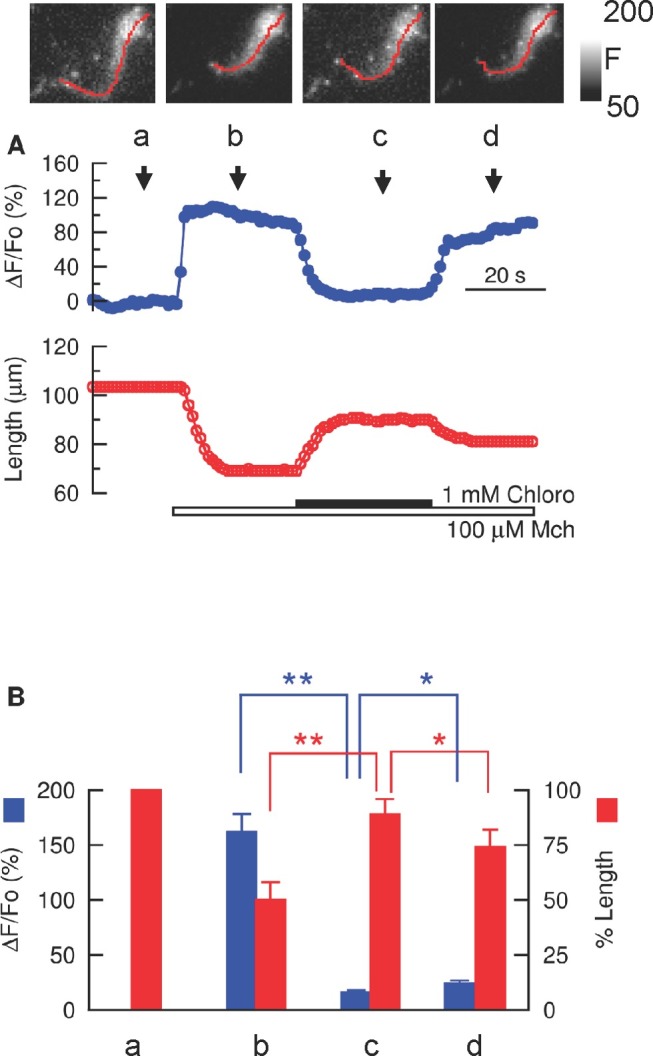
Bitter tastants reverse Mch-induced increase in [Ca^2+^]_i_ and cell shortening in mouse ASM. (A) Time course of the effect of chloro (1 mM) on a 100 µM Mch-induced increase in [Ca^2+^]_i_ (represented as ΔF/F_0_ integrated over the entire cell) and cell shortening. Images show the changes in [Ca^2+^]_i_ displayed as fluorescence intensity (rather than ΔF/F_0_ to aid visualization). Cell length is indicated by the red lines. Images were taken at the time indicated on the time course of [Ca^2+^]_i_ (upper panel). (B) Relationships between [Ca^2+^]_i_ (left axis, blue bars) and cell length (right axis, red bars) in response to Mch and Chloro. The letters correspond to the time shown in the upper panel in (A) (*n* = 23 cells, mean ± SEM; *p<0.05, **p<0.01 using two-tailed Student's *t*-test). ΔF/F_0_ is zero by definition at *a*, so no blue bar is present at a.

Bitter tastants at µM levels can modestly increase [Ca^2+^]_i_ in resting cells, but this raises a conundrum as they also can fully relax airways precontracted by bronchoconstrictors [6],[11]. In light of the fact that an increase in [Ca^2+^]_i_ is the primary signal for contraction in all smooth muscle, we explored how bitter tastants affect [Ca^2+^]_i_ evoked by bronchoconstrictors. To better quantify these effects, we measured ASM Ca^2+^ response and cell shortening at the same time. The cells were stimulated with methacholine (Mch), a stable analogue of acetylcholine, which is the major neurotransmitter in parasympathetic nerves. As expected, Mch (100 µM) rapidly increased [Ca^2+^]_i_ as fluo-3 fluorescence increased by 162%±26% (ΔF/F_0_), and concurrently caused cell shortening by 49%±8% (*n* = 21; Figure 2A and 2B). Strikingly, chloroquine (1 mM) almost completely reversed this [Ca^2+^]_i_ increase (i.e., bringing [Ca^2+^]_i_ down to a level only 15%±2% higher than pre-stimulation levels, *n* = 12, *p*<0.01 Mch versus Mch+chloroquine). The reversal of the increase in [Ca^2+^]_i_ was closely associated with relaxation in ASM cells from both mouse (back to 89%±7% of the pre-stimulation length; Figure 2B and Video S1) and human (back to 94%±5% of the control length; Figure S2A). Denatonium (1 mM) generated similar effects on [Ca^2+^]_i_ and cell shortening in response to Mch in mouse ASM cells (*n* = 9).

**Figure 3:**
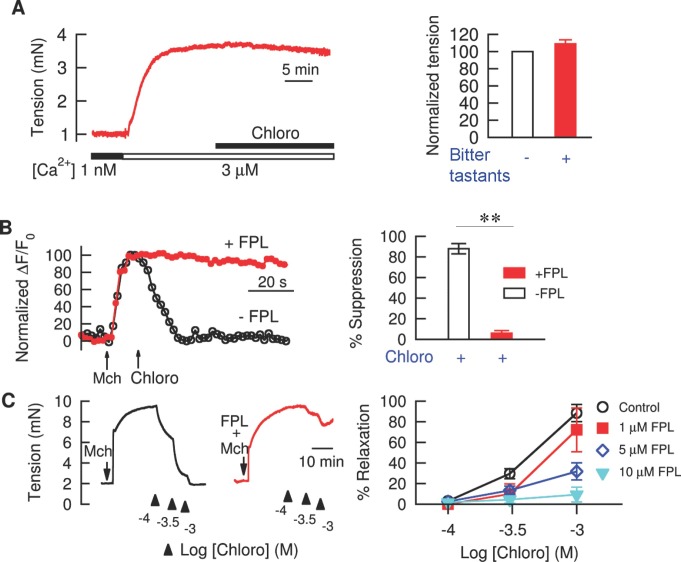
Suppression of [Ca^2+^]_i_ by inhibiting L-type VDCCs is necessary for bitter tastant-induced bronchodilation of Mch precontracted mouse airways. (A) Clamping [Ca^2+^]_i_ prevented bitter tastants from causing bronchodilation. Mouse airway rings were permeabilized with α-toxin (see Method), and extracellular [Ca^2+^]_i_ was set at 1 nM and then switched to 3 µM as indicated under the trace. Seven individual experiments (two for denatonium, two quinine, and three chloro all at 1 mM) show responses similar to that shown on the left, so the results were pooled and displayed on the right. Each ring's normalized tension is its tension at the experiment's end divided by its tension just prior to application of bitter tastants, times 100. (B) FPL 64176 (FPL), an L-type VDCC agonist, prevented chloro from reversing the [Ca^2+^]_i_ rise induced by 100 µM Mch. Left panel: a typical time course; ΔF/F_0_ for each curve is scaled to have a value of 100 at the peak before chloro is added. Right panel: average results of 16 cells. The values are represented as (ΔF/F_0_ at the peak after Mch−ΔF/F_0_ at 30 s after chloro)/(ΔF/F_0_ at the peak after Mch−ΔF/F_0_ at basal)×100 (i.e., the decrease due to chloro divided by the increase due to Mch). **p<0.01, control versus +FPL. (C) FPL dose-dependently reversed chloro-induced bronchodilation (using tension as a proxy measure) in Mch precontracted airways (*n* = 5–7 independent experiments). Data on the right panels are mean ± SEM. % relaxation = tension decrease due to chloro divided by tension increase due to Mch, ×100. The tension decrease at each concentration of chloro is measured once the tension stabilizes. The tension decrease at each increased concentration is always measured relative to the peak tension (i.e., it is total decrease, not the incremental decrease due to the additional chloro which was added).

The inverse relationship between changes in [Ca^2+^]_I_ and the resulting cell length (i.e., lowering [Ca^2+^]_i_ results in cell lengthening) in response to bitter tastants suggests that bitter tastants reduce [Ca^2+^]_i_, leading to bronchodilation. If this is the case, one would expect that bitter tastant-induced bronchodilation could be prevented if [Ca^2+^]_i_ was clamped to a physiologically high level. To test this possibility, we used staphylococcal α-toxin (16,000 units/ml) to make the ASM membrane permeable to ions such that the intracellular [Ca^2+^]_i_ could be controlled at will. A major advantage of using this toxin is that it does not damage the cells; thus signaling processes such as the G-protein-coupled receptor mediated signaling remain intact [23]. As shown in Figure 3A, raising [Ca^2+^]_i_ to 3 µM caused a robust increase in tension in mouse airway. More importantly, at this fixed [Ca^2+^]_i_ level, denatonium, chloroquine and quinine (all at 1 mM) failed to relax ASM in the time frame they would have in Mch contracted airways without α-toxin treatment. Therefore, clamping [Ca^2+^]_i_ at µM levels can prevent bitter tastant-induced bronchodilation, strongly arguing that reduction of [Ca^2+^]_i_ by bitter tastants is necessary for their relaxation action. These results further imply that a decrease in Ca^2+^ sensitivity is probably not a major mechanism underlying bitter tastant-induced bronchodilation.

### The Prominent Role of L-type Ca^2+^ Channels in Mediating Mch-Induced Contraction

Having established that the suppression of [Ca^2+^]_i_ is necessary for bitter tastant-induced bronchodilation, we next addressed the molecule(s) that bitter tastants act on to reduce [Ca^2+^]_i_. Before addressing this critical question, it is appropriate to determine the Ca^2+^ pathways underlying Mch-induced contraction because a controversy persists [24]. Mch activates both the M3 muscarinic acetylcholine receptor (M3R)-Gq-PLCβ-IP3 pathway and the M2 muscarinic acetylcholine receptor (M2R)-Gi/o pathway to raise [Ca^2+^]_i_ by releasing Ca^2+^ from internal stores and inducing Ca^2+^ influx from the extracellular space respectively [25],[26]. It has also been suggested that Ca^2+^ release from the internal stores contributes to the early phase of Mch-induced contraction, and Ca^2+^ influx is required to sustain elevated [Ca^2+^]_i_ and contraction. Indeed, we found that the sustained contraction by Mch in mouse ASM is largely dependent on Ca^2+^ influx (Figure S3). However, the route of Ca^2+^ influx upon muscarinic receptor activation among different species is highly debatable [24]. Many studies suggest that L-type Ca^2+^ channels are the major path of the Ca^2+^ influx for contraction [27]–[32]. To determine the role of this channel in mediating Mch-induced contraction in mouse airways, we examined whether L-type channel specific blockers inhibit Mch-induced contraction and the rise in [Ca^2+^]_i_. Previous studies showed that diltiazem, a well-known L-type Ca^2+^ channel blocker that belongs to the benzothiazepine class, dose-dependently reverses Mch-induced contraction when administrated after Mch's response reaches a plateau [31]–[33]. We therefore examined whether this blocker produces similar inhibition of Mch-induced airway force generation in mouse and human airways. Figures S2B and S4A show that diltiazem dose-dependently inhibited Mch-induced contraction of airways from both species, and, moreover, it reversed the Mch-induced increase in [Ca^2+^]_i_ by 90.2%±2.9% in single isolated mouse ASM cells (*n* = 12 cells). To further examine whether diltiazem inhibits muscarinic receptor-mediated contraction, we treated the airways with diltiazem at different concentrations before Mch administration. Since a unique feature of diltiazem in inhibiting L-type Ca^2+^ channels is its use-dependence of action (i.e., it more likely binds to, and therefore blocks, channels as they open), in this series of experiments we challenged the airways twice with KCl to activate the Ca^2+^ channels. As shown in Figure S4B, under this condition, diltiazem dose-dependently suppressed Mch-induced contraction and at 100 µM it inhibited the force by 85%±6% (*n* = 5). To directly demonstrate that diltiazem inhibits L-type Ca^2+^ channels, we studied the effect of diltiazem on L-type Ca^2+^ currents with patch-clamp recording. We found that diltiazem dose-dependently reduced depolarization-induced L-type Ca^2+^ currents at concentrations over the same range as that which blocked contraction (Figure S4C).

Dihydropyridines (e.g., nisoldipine, nifedipine, and isradipine) are another well-known class of L-type Ca^2+^ channel blockers [34]–[36]. Previously we found that dihydropyridines can inhibit L-type Ca^2+^ current in mouse ASM [17]. To further establish the role of this class of Ca^2+^ channels in mediating Mch-induced contraction, we assessed the effect of nisoldipine on the contraction evoked by Mch. It is known that dihydropyridines bind stronger to inactivated Ca^2+^ channels, thus displaying a so-called voltage dependent inhibition [37]. Therefore, in this series of experiments, to facilitate the interaction between nisoldipine and L-type Ca^2+^ channels, we examined the effect of nisoldipline on Mch-induced contraction in the presence of 20 mM KCl to modestly depolarize the membrane. Under this condition, 1 µM nisoldipine inhibited Mch-induced contraction by 74%±8% (*n* = 5; Figure S4D). This result is similar to the inhibition of this blocker on carbachol-induced contraction in rat ASM, when it is similarly depolarized [29]. Hence, with specific L-type Ca^2+^ channel blockers of distinct structures we have established that L-type VDCCs are the major contributor to Ca^2+^ influx and sustained contraction in response to Mch in mouse airways.

### Bitter Tastants Inhibit L-Type VDCCs to Decrease [Ca^2+^]_i_ Evoked by Bronchoconstrictors

Given the prominent role of L-type VDCCs in Mch-induced sustained contraction in mouse airways, and our and others' findings that bitter tastants reverse Mch-induced sustained contractile [6],[11], we hypothesized that bitter tastants inhibit L-type VDCCs, leading to relaxation of airways precontracted by Mch. To test this possibility, we investigated whether the L-type VDCC agonist FPL 64176 [38],[39] can prevent the inhibitory effect of bitter tastants on the Mch-induced [Ca^2+^]_i_ rise and contraction. At the single cell level, 10 µM FPL prevented chloroquine from reducing the [Ca^2+^]_i_ increase caused by Mch (Figure 3B). At the tissue level, FPL prevented chloroquine from relaxing Mch precontracted mouse ASM in a dose-dependent manner (Figure 3C). These results suggest that bitter tastants inhibit L-type VDCCs, which in turn leads to a decrease in [Ca^2+^]_i_ and resulting bronchodilation.

### Bitter Tastants Reverse the [Ca^2+^]_i_ Rise and Contraction Evoked by Depolarization-Induced Activation of L-Type VDCCs

**Figure 4:**
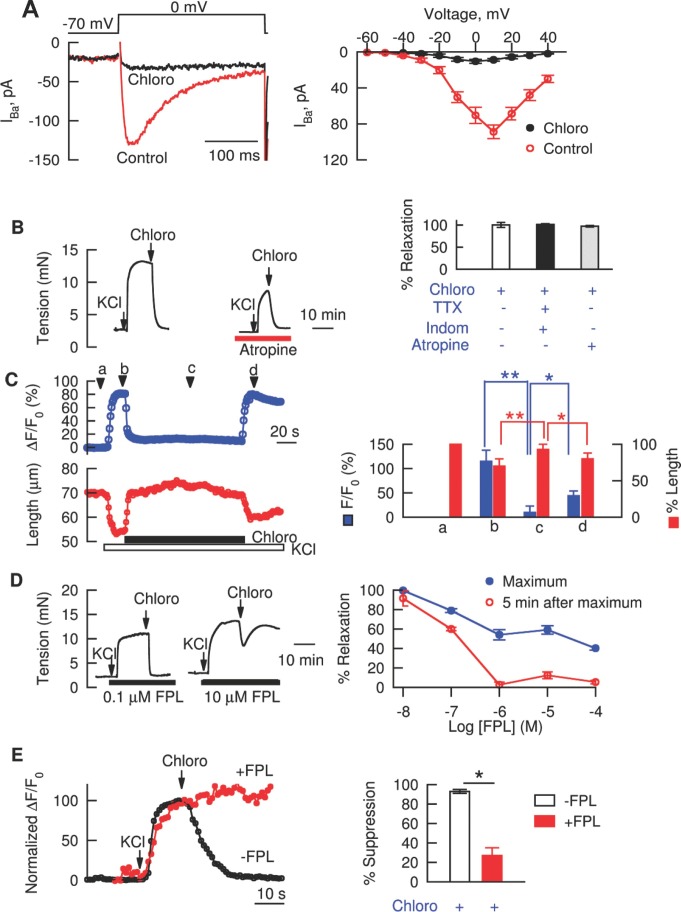
Bitter tastants block L-type VDCCs. (**A**) Chloro blocked L-type VDCC currents. Left panel displays patch clamp recordings of L-type Ca^2+^ currents in response to a voltage pulse from −70 mV to 0 mV in the control and in the presence of 1 mM chloro, and the right panel the effect of chloro on the current-voltage (I–V) relationship of the Ca^2+^ current (*n* = 5). Ba^2+^ was used as a charge carrier, and the peak current was used to construct the I–V relationship. Note that the high voltage threshold for activation seen in the I–V relationship, and its sensitivity to FPL and nifedipine [17] indicate these Ca^2+^ currents resulted from the opening of L-type VDCCs. (B) Bitter tastants relaxed KCl-induced contraction of mouse airways. The left panel shows representative force recordings in response to 60 mM KCl followed by 1 mM chloro in the presence of 100 nM atropine and in its absence. The right panel shows the mean values of the relaxation of KCl-induced contraction by 1 mM chloro in the control (*n* = 9), in the presence of 100 nM atropine (*n* = 16), or in the presence of 1 µM tetrodotoxin (TTX) and 1 µM indomethacin (Indom) (*n* = 6). Note that the inhibition of chloro in the presence of 100 nM atropine was calculated relative to the atropine-resistant contraction in response to KCl. Not shown: 1 mM denatonium relaxed airways precontracted by 60 mM KCl by 97%±4% (*n* = 5 independent experiments). (C) Relationship between [Ca^2+^]_i_ and cell length in response to 60 mM KCl and 1 mM chloro. Left panel shows the time course of concomitant changes in [Ca^2+^]_i_ and cell length and the right the mean ± SEM (*n* = 15 cells) at the four time points marked on the left. *p<0.05; **p<0.01. (D) FPL 64176 (FPL) dose-dependently inhibited chloro-induced bronchodilation of KCl precontracted airways. Left panel shows two representative recordings, and the right panel the means ± SEM (*n* = 5–7 independent experiments). 60 mM KCl and 1 mM chloro were used. Given the non-monotonic nature of the relaxation (left), both the greatest reduction in force after chloro (i.e., maximum) and the force reduction 5 min after chloro were measured and divided by the peak force- resting force before application of chloro. (E) FPL inhibited chloro-induced suppression of the rise in [Ca^2+^]_i_ produced by KCl. Left panel shows original recordings of Ca^2+^ responses and the right panel the mean ± SEM (*n* = 28 without FPL, *n* = 16 with FPL). The values represent as (ΔF/F_0_ at the peak after KCl−ΔF/F_0_ at 30 s after chloro)/(ΔF/F_0_ at the peak after KCl−ΔF/F_0_ at basal)×100.

To directly examine the inhibitory role of bitter tastants on L-type VDCCs, we studied the effect of bitter tastants on L-type VDCC currents using patch clamp recording. Figure 4A shows two representative traces of L-type Ca^2+^ currents evoked by a depolarizing pulse to 0 mV from a holding potential of −70 mV (left panel) and mean peak currents at different depolarizing voltages (right panel) before and after 1 mM chloroquine. As is evident, chloroquine inhibited the L-type Ca^2+^ current when depolarizing voltages are between −30 mV and +40 mV.

To study whether this inhibition by chloroquine of L-type VDCCs could produce relaxation, we evaluated the effect of this bitter tastant on the depolarization-induced increase in [Ca^2+^]_i_ and contraction. KCl is a standard and common reagent used to study cellular processes mediated by depolarization. In airways, depolarization is expected to not only activate VDCCs in ASM but also those in cholinergic nerves as well (leading to release of Ach). Indeed, in ferret and pig, KCl activates both mechanisms to cause airway contraction [40],[41]. However, the Ach release mechanism does not operate in dog and rabbit airway, as demonstrated by several reports that showed that atropine, a muscarinic receptor antagonist, had no measureable effect on the magnitude of tension generated by high K^+^ in these species [42]–[44]. Therefore to determine which mechanisms are activated by KCl in mouse airways, we examined the influence of atropine on KCl-induced contraction. Atropine dose-dependently inhibited Mch-induced contraction of mouse airways, and at concentrations greater than 100 nM it fully blocked the contraction (Figure S5A). These results confirm the efficacy of atropine in inhibiting muscarinic receptors in mouse airways. We further found that atropine (100 nM) reduced KCl (60 mM)-induced contraction to 58%±2.5% of the time matched control (Figure S5), implying that KCl does activate VDCCs in both ASM and cholinergic neurons to cause airway contractions via a combined effect.

Although L-type VDCCs are the major Ca^2+^ channel for Ca^2+^ influx upon depolarization in ASM, and mouse ASM cells exhibit only L-type Ca^2+^ currents [17], it is possible that KCl-induced contraction might involve Rho and Rho kinase via a Ca^2+^-independent mechanism [45]. To examine this possibility, we studied the effect of extracellular Ca^2+^ on the KCl-induced increase in [Ca^2+^]_i_ and contraction. In Ca^2+^ containing medium, KCl (60 mM) induced a marked increase in [Ca^2+^]_i_ (Figure S6A) in isolated ASM cells and airway contraction (Figure S6B). Yet in Ca^2+^ free medium, the same KCl failed to cause any increase in [Ca^2+^]_i_ or a significant contraction (Figure S6B and S6C), consistent with published results in mouse and rat ASM [46],[47]. These results indicate that KCl depolarizes the membrane, leading to a rise in [Ca^2+^]_i_ and a resultant contraction in mouse airway. They also demonstrate that Ca^2+^ influx is necessary to produce the KCl-induced contraction, and that a Ca^2+^-independent mechanism (such as the suggested Rho and Rho kinase pathway) [45],[48],[49] is not sufficient (if needed at all) to produce contraction.

To further establish the role of L-type Ca^2+^ channels in the KCl-induced rise in [Ca^2+^]_i_ and contraction, we investigated the influence of diltiazem on these two effects of KCl. We found that 100 µM diltiazem pretreatment reduced the KCl-induced increase in ΔF/F_0_ from 122%±19% to 16.8%±10% in isolated ASM cells (*n* = 9; Figure S6C); it also reversed the KCl-induced contraction by 93.1%±4.8% in airway tissue (*n* = 6; Figure S6D). Therefore, in mouse ASM, high KCl seems to increase [Ca^2+^]_i_ and cause contraction by depolarizing the membrane and activating L-type VDCCs.

Considering the action of KCl as just described (Figures S5 and S6), we reasoned that bitter tastants would be able to relax airways precontracted by KCl if bitter tastant's inhibition of L-type Ca^2+^ channels underlies its relaxation of airways pre-contracted by Mch (Figure 3). Indeed, we found that a 60 mM KCl-induced contraction in mouse and human airways was fully reversed by either chloroquine (1 mM) or denatonium (1 mM) (Figures 4B and S2C). This reversal is due, at least in part, to a direct inhibition of VDCCs in ASM because (1) in the presence of 100 nM atropine, chloroquine can fully block atropine-resistant contraction (Figure 4B), and (2) when nerve action potentials were blocked by 1 µM tetrodotoxin, a voltage-dependent Na^+^ channel blocker, and arachidonic acid metabolism was inhibited by 1 µM indomethacin, a nonselective inhibitor of cyclooxygenase, the bitter tastants still fully relaxed airways precontracted by KCl (Figure 4B). Similar to their effects on Mch-induced responses (Figure 2B), chloroquine reversed the KCl-induced increase in [Ca^2+^]_i_ and shortening of isolated single ASM cells (Figure 4C; *n* = 7). Moreover, FPL dose-dependently reversed chloroquine-induced relaxation in ASM pre-contracted by KCl (60 mM; Figure 4D), and prevented the reduction of [Ca^2+^]_i_ by chloroquine in cells stimulated by KCl (Figure 4E).

### Gβγ Activation Mediates Bitter Tastant Suppression of the Rise in [Ca^2+^]_i_ Evoked by Activation of L-Type VDCCs

**Figure 5:**
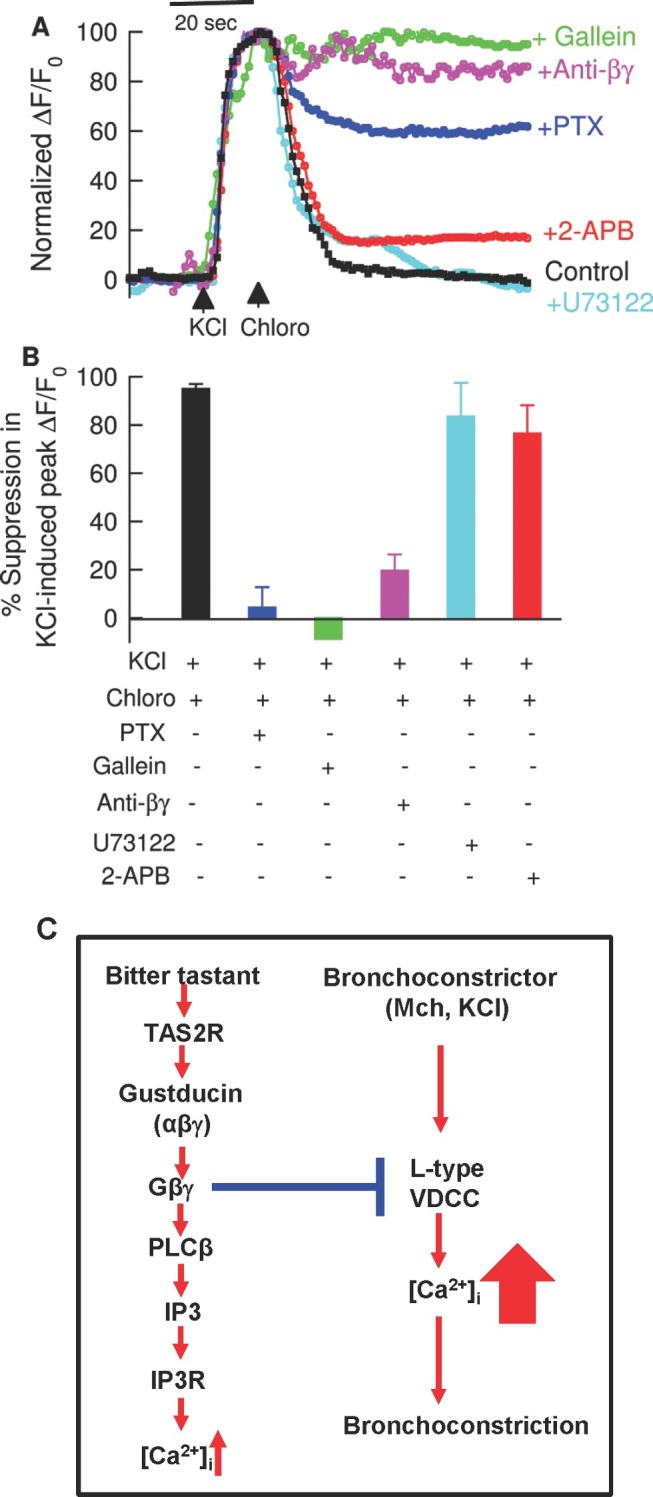
Bitter tastants inhibit L-type VDCCs via a Gβγ dependent process. (A) Representative recordings of changes in [Ca^2+^] in response to 60 mM KCl followed by chloro (I mM) with and without pretreatment with PTX (1 µg/ml), gallein (1 µM), anti-βγ blocking peptide (1 µM), U73122 (3 µM), and 2-APB (50 µM). The application protocols for these compounds were the same as in the experiments in Figure 1C. All data were scaled to have a maximum of 100 and aligned at the time point when KCl was administrated. (B) Effects of compounds listed in *A* on chloro-induced suppression of KCl-induced increase in [Ca^2+^]_i_. The values were calculated the same as in Figure 4E. Compared to the control (i.e., chloro alone after KCl, black filled bar), *p*<0.0001 for PTX, gallein, and anti-βγ; and *p*>0.05 for U73122 and 2-APB. Data are shown as mean ± SEM (*n* = 12–38 cells). (C) A model for TAS2R signaling and bitter tastant-induced bronchodilation. We propose that bitter tastants activate the canonical TAS2R signaling cascade; this modestly increases [Ca^2+^]_i_ in resting cells but exerts no significant effect on resting tone. On the other hand, activation of TAS2Rs activates gustducin and release Gβγ, which turns off L-type VDCCs that are pre-activated by bronchoconstrictors, leading to bronchodilation.

To address the signaling basis underlying bitter tastant inhibition of L-type VDCCs, we studied the impact of perturbing TAS2R signaling on bitter tastant-induced reversal of the [Ca^2+^]_i_ increase in response to KCl in isolated single ASM cells. Pretreatment with PTX at 1 µg/ml for 6–8 h prevented chloroquine-induced reversal of the KCl-induced increase in [Ca^2+^]_i_, as did gallein (20 µM) and anti-βγ, a Gβγ blocking peptide (1 µM) (Figure 5). However, U73122 and 2-ABP, at the concentrations that block the bitter tastant-induced increase in [Ca^2+^]_i_ in resting cells (Figure 1), failed to alter chloroquine's ability to reverse a KCl-induced increase in [Ca^2+^]_i_ (Figure 5). These results indicate that activation of Gβγ but not PLCβ and IP3R is required for bitter tastant-induced inhibition of L-type VDCCs.

## Discussion

Our results demonstrate that bitter tastant's reversal of the rise in [Ca^2+^]_i_ evoked by bronchoconstrictors is required for its bronchodilation effect. They also reveal that bitter tastants can generate different and opposing Ca^2+^ signals depending upon the cellular environment. When administered alone to ASM cells at rest, bitter tastants activate the canonical TAS2R signaling pathway to modestly raise [Ca^2+^]_i_ (Figure 5C) without affecting the contraction. Yet when applied in the presence of the bronchoconstrictors Mch and KCl, they inhibit L-type VDCCs, leading to a reversal of both the evoked [Ca^2+^]_i_ rise and the contraction (Figure 5C). Remarkably, both types of Ca^2+^ signals require Gβγ, while only the increase in resting [Ca^2+^]_i_ depends on PLCβ2 activation and IP3 generation.

Bitter taste receptors (35 in mouse and 25 in human) belong to seven transmembrane domain G-protein-coupled receptors. Long thought to only be expressed in the epithelium cells of the taste buds of the tongue, recent studies have revealed that these receptors also express in several extraoral tissues including brain, testis, immune cells, gastrointestinal tract, and respiratory system [50]–[57]. In airways, these receptors are found to be expressed in ciliated epithelial cells and nasal solitary chemosensory cells [51],[52]. Deshpande et al. [6] reported that multiple TAS2Rs can be detected in cultured human ASM cell lines. In this study, for the first time, to our knowledge, we found that this class of G-protein-coupled receptors is expressed in native mouse ASM cells. Specifically, we determined that TAS2R107, to which both chloroquine and denatonium are ligands, localizes in the cell periphery, a location well suited for mediating bronchodilation in response to these two bitter tastants. We cannot rule out that other types of TAS2Rs also contribute to the bronchodilation induced by chloroquine and denatonium, since both of them can activate multiple mouse and human TAS2Rs [58]. Nevertheless, these ligands do activate TAS2R signaling transduction, resulting in a bronchodilation effect, because pharmacological blocking of multiple downstream components of bitter taste receptors can prevent chloroquine and denatonium-induced cellular responses (Figures 1C, 5A, and 5B). It is also worth noting that chloroquine, denatonium, and all the bitter tastants examined so far are not endogenous ligands for bitter taste receptors. Hence a major question remains as to whether bitter taste receptors in ASM cells have physiological ligands. Interestingly, a recent study revealed that acyl–homoserine lactones, quorum-sensing molecules for Gram-negative pathogenic bacteria, can activate bitter tastant receptors in nasal solitary chemosensory cells to evoke trigeminally mediated reflex reactions, which may trigger an epithelial inflammatory response before the bacteria reach population densities capable of forming destructive biofilms [52],[53]. It would be of great interest and significance to investigate whether these quorum-sensing molecules can activate bitter taste receptors in ASM to induce bronchodilation.

This study revealed two major differences in Ca^2+^ signaling compared to the study by Deshpande et al. [6]. First, these authors reported that bitter tastant increased [Ca^2+^]_i_ to a level comparable to bronchoconstrictors. In freshly isolated ASM, we found that bitter tastants only modestly increase [Ca^2+^]_i_ to a level much lower than that produced by bronchoconstrictors. Second, Deshpande et al. [6] reported that bitter tastants generate local Ca^2+^ events. However, in freshly isolated ASM, we found that bitter tastants do not increase local Ca^2+^ releases such as Ca^2+^ puffs and Ca^2+^ sparks. A reason for these two discrepancies may be that Deshpande et al.'s studies were conducted in cultured ASM cell lines; compared to freshly isolated ASM, these cells display a different phenotype by altering the expression of receptors, ion channels, and contractile proteins [13],[14]. The aforementioned two differences and another difference in which we found that bitter tastants do not activate large-conductance Ca^2+^-activated K^+^ channels strongly argue that bitter tastant-induced bronchodilation is highly unlikely to result from the generation of local Ca^2+^ events, which in turn activate large-conductance Ca^2+^-activated K^+^ channel and hyperpolarize the membrane as proposed [6].

Since bitter tastants relax precontracted airways [6],[11],[15],[16], it is imperative to use a similar stimulating paradigm in order to understand the underlying mechanism of this relaxation. By simultaneously measuring [Ca^2+^]_i_ and cell shortening, we found that bitter tastant's ability to reverse the increase in [Ca^2+^]_i_ caused by bronchoconstrictors is the underlying signal producing the bronchodilation. Three lines of evidence support this conclusion. First, in the presence of bronchoconstrictors, bitter tastants lowered [Ca^2+^]_i_ while at the same time relaxing the precontracted cells, and this response was reversible. Second, clamping intracellular [Ca^2+^]_i_ to levels produced by the bronchoconstrictors (low µM) prevented bitter tastants from relaxing airways. Third, enhancing and blocking Ca^2+^ influx via L-type Ca^2+^ channels can oppositely regulate the relaxation mediated by bitter tastants. These results reinforce the idea that [Ca^2+^]_i_ is the critical signal governing ASM contractility.

The opposing Ca^2+^ signals mediated by Gβγ upon activation of TAS2Rs revealed in this study are unique. It is expected that gustducin Gβγ activates PLCβ to generate IP3 and release Ca^2+^ from endo/sarcoplasmic reticulum to raise [Ca^2+^]_i_ in ASM cells. But, unexpectedly, gustducin Gβγ also suppresses Ca^2+^ signaling mediated by Mch, which largely activates M3R, a Gq family receptor. In general, Gβγ from the G_i_/G_o_ family (to which TAS2Rs belong) tends to potentiate, rather than, inhibit the Ca^2+^ responses caused by the Gq family [59],[60]. It remains to be determined whether the inhibition of Ca^2+^ signaling by TAS2R activation is Gβγ isoform specific. Since Gβγ also mediates the ASM contractions induced by activation of M2R and γ-aminobutyric acid-B receptors [61],[62], our present findings suggested that Gβγ reversal of the rise in [Ca^2+^]_i_ caused by bronchoconstrictors is isoform specific, and is likely via Gβ3γ13 dimers, which are released upon activation of TAS2Rs [63]. Further studies using ASM cells with genetic deletions of these isoforms should facilitate studying this possibility. It is worthy of mention that virtually all of the studies of bitter taste signaling in taste buds [7]–[10] and extraoral tissues [51]–[53] have focused on the responses mediated by bitter tastants alone; the opposing Ca^2+^ signaling mediated by Gβγ as revealed in the present study likely operates in these systems when they are stimulated by a combination of bitter tastants and other activators.

L-type VDCCs in smooth muscle can be modulated by a variety of means including phosphorylation and Ca^2+^ [64]–[68]. Yet for the first time, to the best of our knowledge, we show that βγ subunits of G-protein gustducin can inhibit these channels in smooth muscle, extending the similar findings for cloned Cav1.2 in heterologous expression cells and Cav1.1 in skeletal muscle fiber [69],[70]. This interpretation is buttressed by the experiments showing that the contraction mediated by KCl-induced activation of presynaptic Ca^2+^ channels can also be fully blocked by bitter tastants (Figure 4B). What remains unknown is whether Gβγ directly or indirectly inhibits these channels, and the structural basis for this inhibition. Given that Gβγ can directly inhibit K^+^ channels and N-type Ca^2+^ channels in several cell types [71]–[75], it is likely that Gβγ acts on L-type VDCCs in a similar manner.

Gustducin βγ subunits inhibit L-type VDCCs to cause bronchodilation, highlighting the importance of these channels in mediating bronchoconstriction and their potential as a target for bronchodilators. Indeed, L-type VDCCs are expressed in ASM cells and their activation causes these cells to fully contract (Figures 4, S4, S5, S6) [17],[27],[76],[77]. Also, activation of these channels is a major mechanism underlying bronchoconstrictor-induced contraction of different species including airway and human ([25],[27],[30],[32], but see [24]). Moreover, three classes of organic L-type VDCC blockers (i.e., dihydropyridines, phenylalkylamines, and benzothiazepines) are effective in relieving airway spasm in animal models of asthma and in exercise-induced asthmatic patients [78]–[81]. A long-standing puzzle regarding L-type channels in ASM is that clinical trials in the 1980s suggested that antagonists for this channel were of limited use treating asthma in the population as a whole [81],[82]. A potential reason for this enigma may be to some extent related to the mode of action of these blockers. It is known that these classic organic blockers exert their inactivation of L-type VDCCs in a voltage, stimulation, and frequency dependent manner [34],[36],[37]. Interestingly, allergen sensitized guinea-pig and rabbit ASM cells have a more hyperpolarized membrane potential than normal cells [83]. This implies that L-type Ca^2+^ channel blockers (for example, dihydropyridines) would bind more weakly with these channels, thus decreasing the efficacy of these agents to inhibit these channels, should ASM cells from asthma patients have a more negative membrane potential. Bitter tastants, by their ability to inhibit L-type Ca^2+^ channels via activation of gustducin Gβγ, perhaps could circumvent the drawbacks of the currently available L-type Ca^2+^ channel blockers, and thus be a more effective asthma treatment. This is likely given that bitter tastants induce a stronger bronchodilation in both in vitro and in vivo asthmatic mouse models than do β2 agonists [6],[11], the most commonly used bronchodilators for treating asthma and COPD.

Although bitter tastants are promising candidates to be developed as a new class of bronchodilators, and the findings in the present study provide the cellular and molecular rationale for this line of inquiry, we would caution that chloroquine and denatonium examined in this study may not be ideal candidates because of the high concentration (i.e., on the order of 100 µM) needed to fully relax precontracted ASM. This caveat, however, should not dampen enthusiasm for this endeavor as there are many thousands of bitter tastants available from plants and animals, and numerous bitter small molecules synthesized by research laboratories and a variety of companies over the years. In fact, bitter tastants can stimulate bitter taste receptors at concentrations in the nanomolar range: strychnine activates human TAS2R46 with an EC_50_ of 430 nM and aristolochic acid activates human TAS2R43 with an EC50 of 81 nM [84],[85]. Therefore, it is highly likely that bitter tastants with a highly potent bronchodilating action can be discovered. Searching for these bitter tastants is of clinical significance because the current bronchodilators are insufficient for treating severe asthma and many COPD patients. A critical step in identifying highly potent bitter tastants is developing reliable and highly effective screening methodologies. Simultaneous measurements of cell shortening and the [Ca^2+^]_i_ signal (i.e., a decrease of elevated [Ca^2+^]_i_), as developed in the present study, are robust and quantitative and provide a powerful paradigm for identifying potential bronchodilators from among the many bitter tastants available.

## Materials and Methods

### Animal Tissue Handling

Experimental protocols for animal research were approved by the Institutional Animal Care and Use Committees at the University of Massachusetts Medical School (protocol A-1473 to RZG).

### Isolation of Mouse Airway Smooth Muscle Cells

C57BL/6 mice from 7 to 12 wk of age were anesthetized with intraperitoneally injected pentobarbitone (50 mg kg^−1^), and the trachea and mainstem bronchi were quickly removed and placed in a pre-chilled dissociation solution consisting of (in mM): 135 NaCl, 6 KCl, 5 MgCl_2_, 0.1 CaCl_2_, 0.2 EDTA, 10 HEPES, and 10 Glucose (pH 7.3). Tracheas and mainstem bronchi were dissected free from the surface of the connective tissue. The airway tissue was incubated in the dissociation medium containing papain 30 unit/ml, 1 mM DTT, and 0.5 mg/ml BSA, at 35°C for 30 min, and then transferred to a dissociation medium containing 3 unit/ml collagenase F and 0.5 mg/ml BSA, and incubated at 35°C for another 15 min to produce isolated ASM cells. Finally, the tissue was agitated with a fire polished wide-bore glass pipette to release the cells.

### Mouse Airway Smooth Muscle Contraction Bioassay

C57BL/6 mice at 7–12 wk of age were sacrificed and the entire respiratory trees were rapidly removed and immersed in Krebs physiologic solution containing (in mM) 118.07 NaCl, 4.69 KCl, 2.52 CaCl_2_,1.16 MgSO_4_, 1.01 NaH_2_PO_4_, 25 NaHCO_3_, and 11.10 glucose. Trachea and mainstem bronchi were isolated and cut into rings (4 mm in length). The rings were mounted on a wire myograph chamber (Danish Myo Technology), and a PowerLab recording device (AD Instruments) was used to record isometric tension. The ring preparations with zero tension were immersed in 5 ml of Krebs physiologic solution, bubbled with 95% O_2_ and 5% CO_2_ at 37°C. After 10 min equilibration, three stretches (each 2.5 mN) at 5 min intervals were applied to the rings. After these stretches the basal tones of the rings were usually settled at approximately 2 mN. To test the contractile response, each ring was stimulated twice with KCl (60 mM), separated by 20 min, before proceeding to other treatments. The order and treatment time of agonists and antagonists are indicated in the figure captions. In the experiments in Figures 4B, S4B, and S4D, 1 µM tetrotodoxin was added to prevent action potentials of neurons and 1 µM indomethacin to inhibit cyclooxygenase. The force in response to 60 mM KCl in the presence of tetrodotoxin and indomethacin was 95%±7% (*n* = 6) of that in their absence.

### Airway Smooth Muscle Permeabilization

Mouse bronchi rings (4 mm in length) free of connective tissues were incubated for 5 min in HEPES-Tyrode (H-T) buffer which contained 137.0 mM NaCl, 2.7 mM KCl, 1.0 mM MgCl_2_, 1.8 mM CaCl_2_, 10 mM HEPES, 5.6 mM glucose (pH 7.4). The rings were then transferred to and incubated in Ca^2+^-free H-T buffer for 5 min followed by another 5 min in buffer A (30 mM TES, 0.5 mM DTT, 50 mM KCl, 5 mM K_2_EGTA, 150 mM sucrose [pH 7.4]). To skin ASM, the rings were incubated for 45 min with α-toxin (16,000 units/ml) in buffer A at room temperature. After the permeabilization, the rings were treated with 10 µM ionomycin for 10 min to deplete intracellular Ca^2+^ stores.

Skinned airway rings were mounted on the wire myograph chamber and washed two times with pCa 9 solution (20 mM TES, 4 mM K_2_EGTA, 5.83 mM MgCl_2_, 7.56 mM potassium propionate, 3.9 mM Na_2_ATP, 0.5 mM dithioerythritol, 16.2 mM phosphocreatine, 15 units/ml creatine kinase [pH 6.9]). The viability of the skinned muscle rings was examined by stimulation with pCa 4.5 solution (20 mM TES, 4 mM CaEGTA, 5.66 mM MgCl_2_, 7.53 mM potassium propionate, 3.9 mM Na_2_ATP, 0.5 mM dithioerythritol, 16.2 mM phosphocreatine, 15 units/ml creatine kinase [pH 6.9]) followed by the pCa 9.0 solution. The muscles that could generate sustained contraction in response to the pCa 4.5 solution and fully relax when exposed to the pCa 9.0 solution were used for subsequent experiments. To test the effect of bitter tastants, the viable muscle rings were induced to contract by exposure to pCa 5.5 solution followed by the administration of bitter tastants at the concentrations indicated in Figure 3.

### Measurement of Global [Ca^2+^]_i_ and Ca^2+^ Sparks

Fluorescence images using fluo-3 as a calcium indicator were obtained using a custom-built wide-field digital imaging system. The camera was interfaced to a custom made inverted microscope, and the cells were imaged using either a 20× Nikon 1.3 NA for global [Ca^2+^] measurement or a 60× Nikon 1.4 NA oil for Ca^2+^ spark measurement. The 488 nm line of an Argon Ion laser provided fluorescence excitation, with a shutter to control exposure duration, and emission of the Ca^2+^ indicator was monitored at wavelengths >500 nm. The images were acquired at the speed of either 1 Hz for global [Ca^2+^] measurement or 50 Hz for Ca^2+^ spark measurement. Subsequent image processing and analysis was performed off line using a custom-designed software package, running on a Linux/PC workstation. [Ca^2+^]_i_ was represented as ΔF/F_0_×100 with F calculated by integrating fluo-3 over entire cells for global [Ca^2+^] after background correction with areas free of cells, or just the value at the brightest pixel (i.e., epicenter pixel) for Ca^2+^ sparks.

### Patch-Clamp Recording

Membrane currents were recorded with an EPC10 HEKA amplifier under perforated whole-cell patch recording configuration. The extracellular solution contained (in mM): NaCl 126, tetraethylammonium Cl 10, BaCl_2_ 2.2, MgCl_2_ 1, Hepes 10, and glucose 5.6 (pH adjusted to 7.4 with NaOH). The pipette solution contained (in mM): CsCl 139, MgCl_2_ 1, Hepes 10, MgATP 3, Na_2_ATP 0.5 (pH adjusted to 7.3 with KOH); amphotericin B was freshly made and added to the pipette solution at a final concentration of 200 µg/ml. Whole-cell Ba^2+^ currents were evoked by step depolarization with 300 ms duration every 10 s from a holding potential of −70 mV at a 10 mV increment (Figure 4A) or with protocol as described in the caption of figure caption (Figure S4C). Currents were leak corrected using a P/4 protocol.

### Measurement of Cell Shortening

Myocytes were placed into a recording chamber superfused with the bath solution for patch clamp experiments at room temperature. Cells loaded with Fluo-3 were imaged using a custom-built wide-field digital imaging system and their lengths were determined using custom software to manually trace down the center of the cell [17].

### Reverse Transcription-PCR to Detect mRNA

The connective tissues in trachea and mainstem bronchi were carefully removed and the ASM were then quickly frozen in dry ice. The total RNA of the ASM was isolated with the TRIzol (Invitrogen) method following the manufacturer's guidelines; and cDNA was synthesized using extracted RNA with an Omniscript Reverse Transcription kit (Qiagen). The specific primers, synthesized by Invitrogen, are listed in Table S1. β-actin was used as a positive control and the absence of DNA as a negative control, and the PCR reactions were carried out in a PCR mastercycler.

### Immunocytochemistry

Mouse ASM cells, isolated as described above and plated onto poly-L-lysine coated coverslips were fixed and permeabilized (0.1 M ethanolamine in PBS plus 0.1% triton X-100 [pH 8]) and then immunolabeled as described previously [86]. Anti-TAS2R107, an affinity purified rabbit polyclonal antibody raised against a peptide mapping within an extracellular domain of mouse TAS2R107, was purchased from Santa Cruz Biotechnology (sc-139175), and purified IgG was used as control.

3D fluorescence imaging was performed on an inverted wide field microscope (Nikon Diaphot 200) with excitation by a 100 W mercury lamp. Images were obtained through a 60× objective and digitally recorded on a cooled, back-thinned CDD camera (Photometrics), with an effective pixel size at the specimen of 83 nm in x-y and a z spacing of 100 nm. This resulted in a 3D stack of approximately 100 image planes for each cell.

The fluorescence images were deconvolved with a constrained, iterative approach [87] originally designed for UNIX systems. The algorithm was rewritten using FFTW, a free, fast Fourier transform library and implemented as a multiuser client/server system on computers running the Fedora operating system (Red Hat), either stand-alone or configured in a Beowulf cluster. Each image was dark current and background subtracted, flat-field corrected, and then deconvolved. After deconvolution images were thresholded to eliminate non-specific binding. Voxels that fell below a threshold were considered to be non-specific bindings and were set to zero; all other voxels remained unchanged. This threshold was derived from analysis of control images containing purified IgG. The intensity which eliminated 99% of the voxels in the control images became the threshold intensity.

### Reagents and Their Application

All chemicals, except fluo-3 (Invitrogen Co), gallein (Tocris Bioscience), anti-βγ blocking peptide (AnaSpec), anti-TAS2R107 (Santa Cruz Biotechnology), and purified IgG (Jackson ImmunoResearch Laboratories) were purchased from Sigma-Aldrich Co. For single cell studies, agonists and antagonists were applied locally to cells via a picospritzer at a constant pressure, so that the duration of its action and concentration could be controlled easily.

### Statistics

Unless stated otherwise, data are reported as mean ± standard error of the mean (SEM) and *n* represents the number of cells or trachea and mainstem bronchi. Statistical analysis of differences was made with Student's paired or unpaired *t*-test and the significance level was set at *p*<0.05.

## Supporting Information

Figure S1 **Bitter tastant chloroquine dose-dependently increased [Ca^2+^]_i_ in resting single cells (A) without a significant effect on the contractility (B) of relaxed mouse airways.** Results are mean ± SEM, (*n* = 5–30 cells in (A) and 7 airway rings in (B)). Dose response curves in (A) were generated on the basis of the responses to single dose administration, while that in (B) was based on accumulative administration. Note that Mch produced much larger responses.
(TIFF)

Figure S2 **Characteristics of [Ca^2+^]_i_ and contractile responses to bitter tastants and diltiazem in human ASM.** (A) Bitter tastants reversed the [Ca^2+^]_i_ rise and cell shortening induced by Mch. Measurements were taken at the steady state levels in response to Mch and chloroquine. The cell length before stimulation was considered as 100%. *p<0.05 paired Student's *t*-test; ***p<0.001; *n* = 6–12. (B) L-type VDCC blocker diltiazem dose-dependently reversed 10 µM Mch-induced contraction (*n* = 5 independent experiments). % relaxation = tension decrease due to diltiazem divided by tension increase due to Mch, times 100. The tension decrease at each concentration of diltiazem is measured once the tension stabilizes. The tension decrease at each increased concentration is always measured relative to the peak tension (i.e., it is total decrease, not the incremental decrease due to the additional diltiazem which was added). (C) Chloroquine (1 mM) and diltiazem (100 µM) relaxed human intrapulmonary bronchi precontracted by 60 mM KCl (*n* = 3–5 independent experiments). % relaxation = tension decrease due to chloroquine divided by tension increase due to Mch, times 100. Bar charts are mean ± SEM. Human lung tissues were obtained (with informed consent) from patients undergoing surgery (lobectomy) for lung cancer at the Department of Surgery and the Department of Pathology at the University of Massachusetts Memorial Medical Center (Worcester). The tumors were identified as non–small cell carcinoma (adenocarcinoma or squamous cell carcinoma). Intrapulmonary airways were dissected out and cleaned free of the connective tissues. These airways were either cut into the rings (4 mM in length) for force measurements the same as for mouse airway tissues, or digested with the same enzymes, dissociation medium, and isolation procedure as for single mouse ASM cells. The experimental protocols on human tissues were approved by the Committee for Protection of Human Subjects in Research at the University of Massachusetts Medical School (protocol 13590 to RZG).
(TIFF)

Figure S3 **Ca^2+^ influx plays a major role in producing and maintaining Mch-induced increases in [Ca^2+^]_i_ and contraction in mouse ASM.** (A) In Ca^2+^ free medium, the tension generated by Mch was less than 20% of that in the presence of extracellular Ca^2+^. ***p<0.001, Student's paired *t*-test, *n* = 9 airway rings for the group with Ca^2+^, and *n* = 10 airway rings for the group without Ca^2+^. (B) Mch increased [Ca^2+^]_i_ much less in Ca^2+^ free medium than in the presence of extracellular Ca^2+^. In the absence of extracellular Ca^2+^, Mch (10 µM) produced different patterns of changes in [Ca^2+^]_i_, so the area under each curve was calculated for 1 min of Mch stimulation and compared between the two conditions (right panel). ***p<0.001 with extracellular Ca^2+^ (*n* = 9 cells) versus without the Ca^2+^ (*n* = 12 cells); Student's unpaired *t*-test. (C) Ca^2+^ stores remained functional in the absence of extracellular Ca^2+^. The cells were placed in the absence of extracellular Ca^2+^ for ∼15 min, and then stimulated with two 10 µM Mch pulses 15 min apart. The chart on the right indicates that two Mch administrations produced comparable Ca^2+^ responses, i.e., Ca^2+^ stores are intact under experimental conditions in the present study. NS, *p*>0.05 for the response in the first pulse of Mch versus that in the second pulse, Student's paired *t*-test, *n* = 10. ΔF/F_0_ for (B) and (C) are the average over the entire cell.
(TIFF)

Figure S4 **Role of L-type Ca^2+^ channel activation in Mch-induced contraction in mouse airway.** (A) Left panel: L-type VDCC blocker diltiazem dose-dependently reversed Mch-induced contraction (using tension as a proxy measure). Right panel: results for *n* = 6 airway rings. % relaxation was calculated the same as in Figure S2B. (B) Diltiazem inhibited Mch- and KCl-induced contraction in a dose-dependent manner. (i) Once equilibrated, airway rings generated stable responses to 60 mM KCl (i.e., K0, K1, and K2), and to 1 µM Mch (i.e., M0 and M1) over a time span longer than 1 hr. (ii, iii, iv) show representative responses to KCl and Mch in the presence of diltiazem at 1 µM, 10 µM, and 100 µM, respectively. Two pulses of KCl (K1 and K2) were administrated before Mch (M1) to facilitate diltiazem inactivation of L-type Ca^2+^ channels. (v) Dose-response curve for diltiazem-mediated inhibition of contraction by KCl. Data are mean ± SEM (*n* = 5); % inhibition = (force by K0 − force by K2)/(force by K0)×100. (vi) Dose-response curve for diltiazem-mediated inhibition of contraction by Mch. Data are mean ± SEM (*n* = 5); % inhibition = (force by M0 – force by M1)/(force by M0)×100. Forces are measured at their peak, excluding the noise spikes due to the washing out of KCl or Mch. (C) Diltiazem inhibited L-type VDCC currents in a dose-dependent manner. Diltiazem was added to the bath solution cumulatively. Once at equilibrium at each level of diltiazem, cells were stimulated with a train of ten voltage pulses from −70 mV to 0 mV (inset) at 10 s intervals. Left panel displays patch clamp recordings of L-type Ca^2+^ currents in the control and in the presence of diltiazem at the given concentration (each in response to the tenth voltage pulse), and the right panel depicts the effect of diltiazem on the peak current at different concentrations. Ba^2+^ was used as the charge carrier. Data are mean ± SEM (*n* = 6); % inhibition = (peak current of the control − peak current at given diltiazem concentration)/peak current of the control ×100 (measured in the tenth voltage pulse). (D) Effect of nisoldipine on Mch-induced contraction. In light of the voltage-dependence of the inhibition of nisoldipine on L-type VDCCs, airway rings were modestly depolarized with 20 mM KCl (red bars) before Mch stimulation. Left panel displays a representative contractile response to 60 mM KCl and 1 µM Mch before and after 1 µM nisoldipine. Right panel shows the mean values (mean ± SEM) of inhibition by 1 µM nisoldipine of contraction evoked by KCl and Mch, respectively. % inhibition = (maximal force at the control − maximal for with nisoldipine)/maximal force at the control ×100.
(TIFF)

Figure S5 **KCl activates VDCCs in cholinergic nerves and ASM.** (A) Atropine dose-dependently inhibited Mch-induced mouse airway contraction. The left panels display representative contractile responses to 3 µM Mch in the absence or the presence of atropine as marked near the traces, and the right panel shows the mean values (mean ± SEM; *n* = 4–8) of inhibition by atropine of contraction evoked by 3 µM Mch. % inhibition = (maximal force from the control − maximal force with atropine)/maximal force from the control ×100. (B) Atropine inhibited KCl-induced mouse airway contraction. The left panel shows a representative tension recording in response to 60 mM KCl before and after 100 nM atropine, as marked beneath. The right panel shows the summarized results as the ratio of the force generated by the second KCl pulse over first KCl pulse (mean ± SEM; *n* = 6 for the time matched controls, *n* = 28 for atropine).
(TIFF)

Figure S6 **KCl activates L-type VDCCs to increase [Ca^2+^]_i_ and cause contraction in mouse ASM.** (A) KCl failed to generate any global [Ca^2+^]_i_ increase in the absence of extracellular Ca^2+^ in isolated single ASM cells. (i) A representative [Ca^2+^]_i_ response to 60 mM KCl in the presence of extracellular Ca^2+^. (ii, iii, iv) three examples showing that the same concentration of KCl did not increase Ca^2+^ in the zero Ca^2+^ medium. This failure was not due to the depletion of intracellular Ca^2+^ stores because 10 µM Mch still induced Ca^2+^ release either as a single peak or as oscillations. Eight cells gave rise to similar responses. ΔF/F_0_ is the average over the entire cell. (B) KCl (60 mM) caused virtually no increase in tension in the absence of extracellular Ca^2+^. The airways were placed in the Ca^2+^ free solution for 15 min before the measurement commenced. Left panel shows a pair of representative recordings and right panel the average results. **p<0.01, Student's paired *t*-test, *n* = 6 independent experiments. (C) KCl (60 mM)-induced increase in [Ca^2+^]_i_ was markedly inhibited by prior application of L-type VDCC blocker diltiazem (100 µM). **p<0.01, Student's paired *t*-test, *n* = 9 for each conditions. (D) Diltiazem (100 µM) relaxed 60 mM KCl-induced contraction of mouse airways. Data are mean ± SEM (*n* = 6 independent experiments), and % relaxation definition and analysis are the same as in Figure S2C.
(TIFF)

Table S1 **Primers for Reverse Transcription-PCR.**
(TIFF)

Movie S1 This clip shows 1 mM chloro reversed the 100 µM Mch-induced increase in [Ca^2+^]_i_ and cell shortening; the first 120 images of this clip were analyzed and plotted in Figure 2A. The images are displayed as fluorescence intensity (rather than ΔF/F_0_) because the cell changes its shape dramatically in response to stimuli (and changing thickness makes ΔF/F_0_ measures misleading).
(MOV)

## Abbreviations

2-APB: 2-aminoethoxydiphenyl borate
ASM: airway smooth muscle
[Ca^2+^]_i_: intracellular Ca^2+^ concentration
COPD: chronic obstructive pulmonary disease
IP3R: inositol 1,4,5-triphosphate receptor
Mch: methacholine
PLCβ: phospholipase Cβ
MR: muscarinic acetylcholine receptor
PTX: pertussis toxin
SEM: standard error of the mean
TAS2R: bitter taste receptor
VDCC: voltage-dependent Ca^2+^ channel
