# Correction: The Cellular and Molecular Basis of Bitter Tastant-Induced Bronchodilation

**DOI:** 10.1371/annotation/7899a865-d68b-45bd-8b9b-ec6f50c9308a

**Published:** 2013-03-15

**Authors:** Cheng-Hai Zhang, Lawrence M. Lifshitz, Karl F. Uy, Mitsuo Ikebe, Kevin E. Fogarty, Ronghua ZhuGe

The following information was missing from the Funding section: This study was supported by NIH grant HL073050 to Mitsuo Ikebe.

