# Supplementary figures and images for: The Cellular and Molecular Basis of Bitter Tastant-Induced Bronchodilation
Source: PLoS Biol. 2013 Mar 5;11(3):e1001501. doi: 10.1371/journal.pbio.1001501 (PMC3589262; doi:10.1371/journal.pbio.1001501)

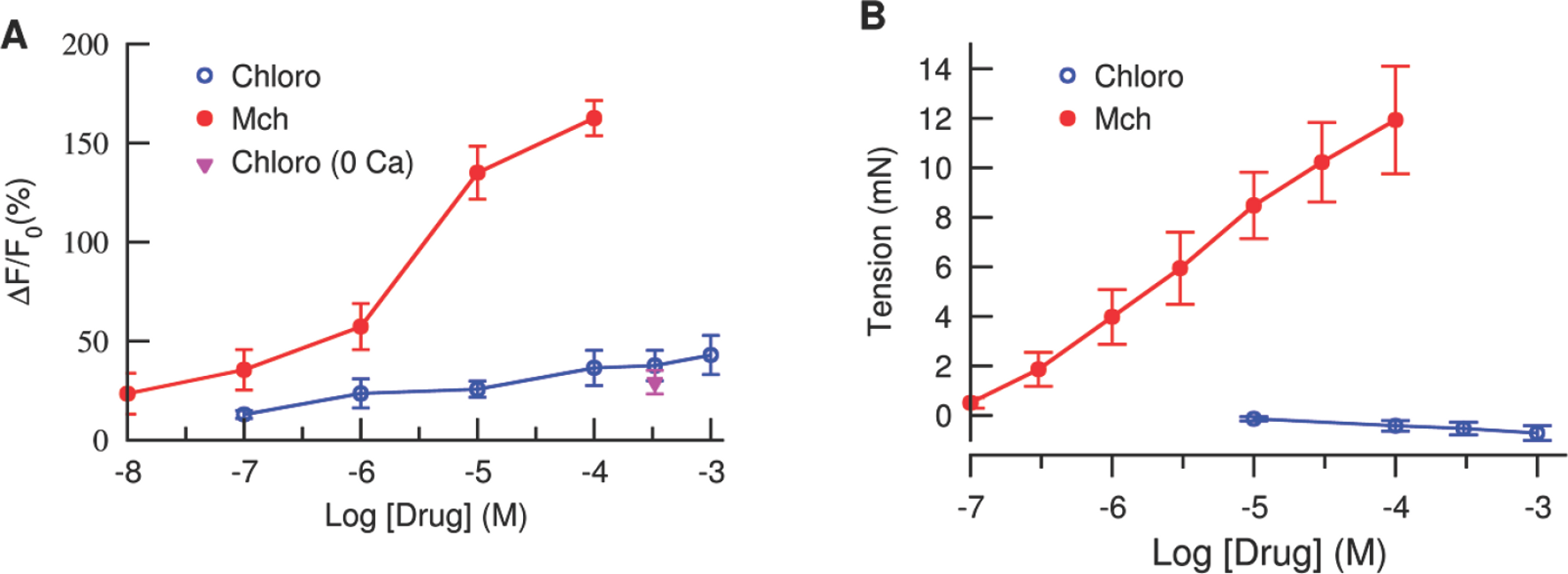

Supplement: Figure S1 — Bitter tastant chloroquine dose-dependently increased [Ca2+]i in resting single cells (A) without a significant effect on the contractility (B) of relaxed mouse airways. Results are mean ± SEM, (n = 5–30 cells in (A) and 7 airway rings in (B)). Dose response curves in (A) were generated on the basis of the responses to single dose administration, while that in (B) was based on accumulative administration. Note that Mch produced much larger responses. (TIFF) [file pbio.1001501.s001.tiff]

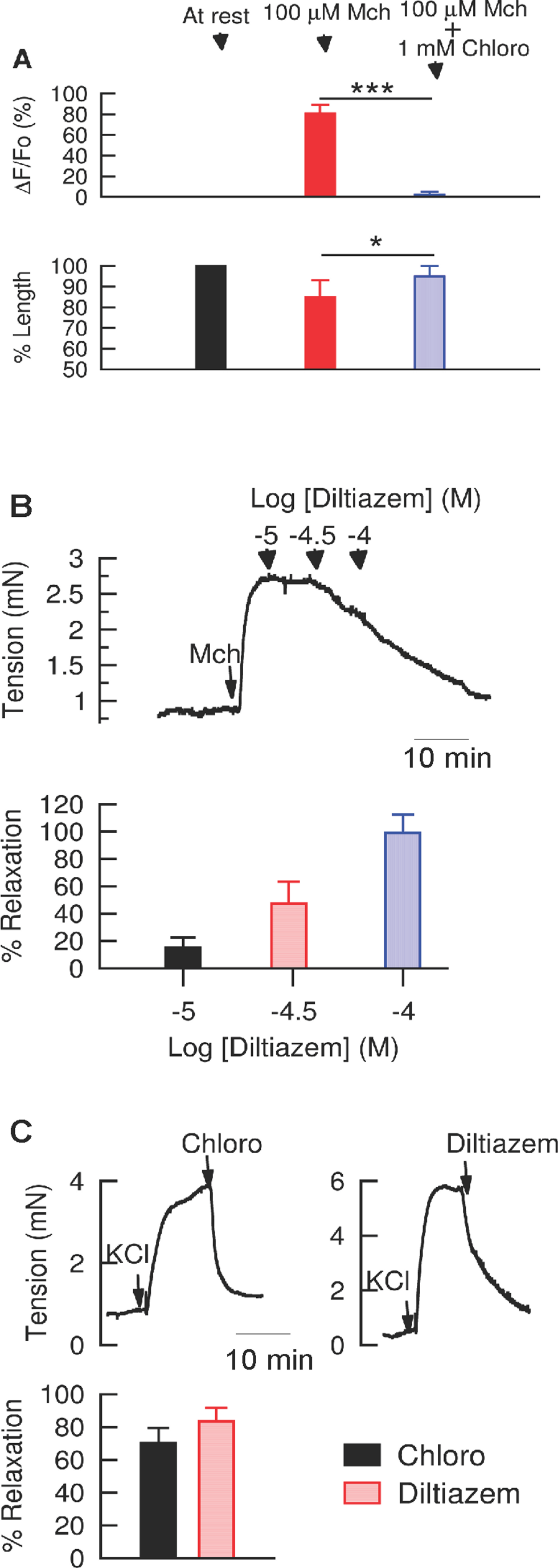

Supplement: Figure S2 — Characteristics of [Ca2+]i and contractile responses to bitter tastants and diltiazem in human ASM. (A) Bitter tastants reversed the [Ca2+]i rise and cell shortening induced by Mch. Measurements were taken at the steady state levels in response to Mch and chloroquine. The cell length before stimulation was considered as 100%. *p<0.05 paired Student's t-test; ***p<0.001; n = 6–12. (B) L-type VDCC blocker diltiazem dose-dependently reversed 10 µM Mch-induced contraction (n = 5 independent experiments). % relaxation = tension decrease due to diltiazem divided by tension increase due to Mch, times 100. The tension decrease at each concentration of diltiazem is measured once the tension stabilizes. The tension decrease at each increased concentration is always measured relative to the peak tension (i.e., it is total decrease, not the incremental decrease due to the additional diltiazem which was added). (C) Chloroquine (1 mM) and diltiazem (100 µM) relaxed human intrapulmonary bronchi precontracted by 60 mM KCl (n = 3–5 independent experiments). % relaxation = tension decrease due to chloroquine divided by tension increase due to Mch, times 100. Bar charts are mean ± SEM. Human lung tissues were obtained (with informed consent) from patients undergoing surgery (lobectomy) for lung cancer at the Department of Surgery and the Department of Pathology at the University of Massachusetts Memorial Medical Center (Worcester). The tumors were identified as non–small cell carcinoma (adenocarcinoma or squamous cell carcinoma). Intrapulmonary airways were dissected out and cleaned free of the connective tissues. These airways were either cut into the rings (4 mM in length) for force measurements the same as for mouse airway tissues, or digested with the same enzymes, dissociation medium, and isolation procedure as for single mouse ASM cells. The experimental protocols on human tissues were approved by the Committee for Protection of Human Subjects in Research at the University of Ma [file pbio.1001501.s002.tiff]

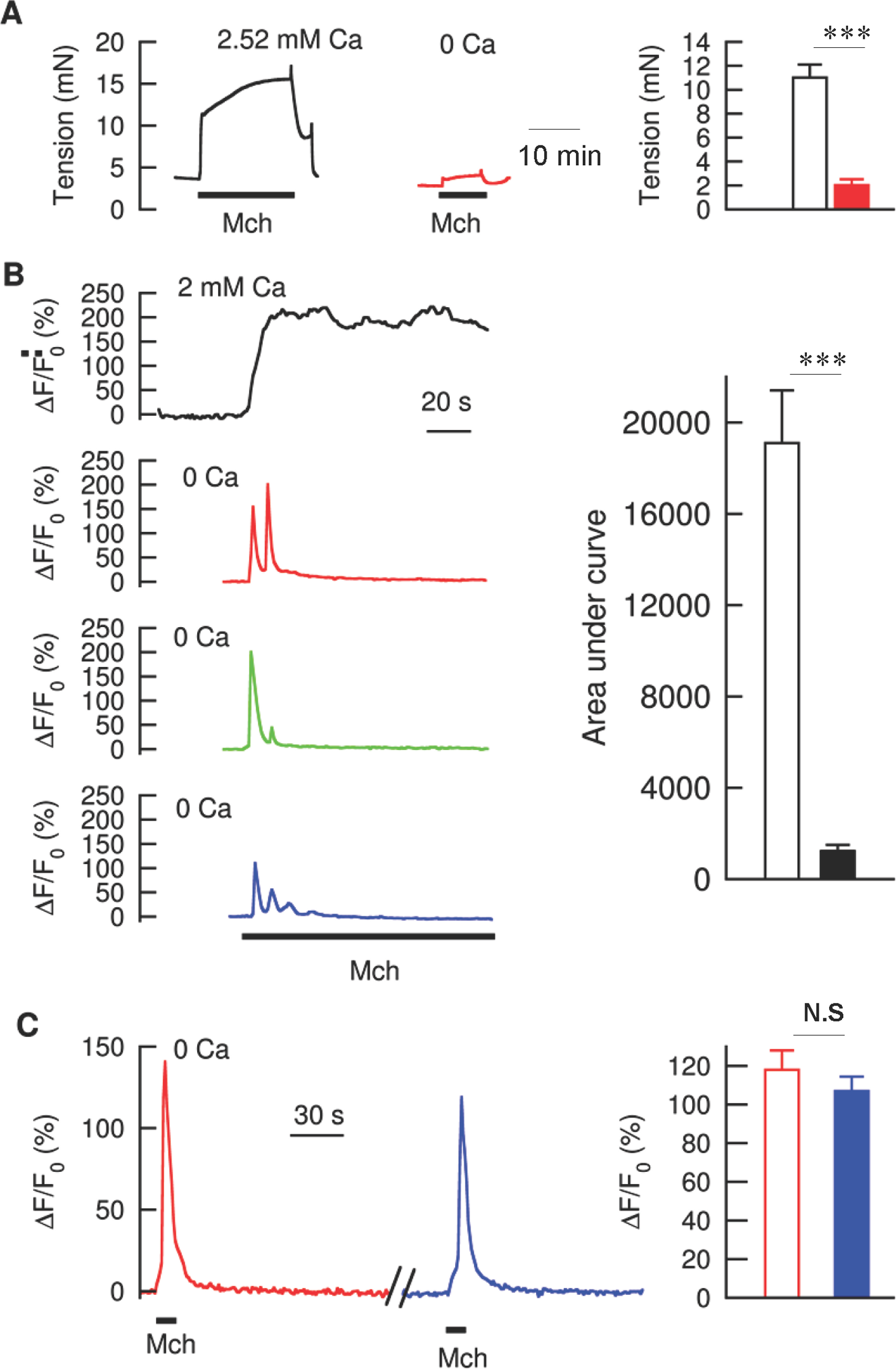

Supplement: Figure S3 — Ca2+ influx plays a major role in producing and maintaining Mch-induced increases in [Ca2+]i and contraction in mouse ASM. (A) In Ca2+ free medium, the tension generated by Mch was less than 20% of that in the presence of extracellular Ca2+. ***p<0.001, Student's paired t-test, n = 9 airway rings for the group with Ca2+, and n = 10 airway rings for the group without Ca2+. (B) Mch increased [Ca2+]i much less in Ca2+ free medium than in the presence of extracellular Ca2+. In the absence of extracellular Ca2+, Mch (10 µM) produced different patterns of changes in [Ca2+]i, so the area under each curve was calculated for 1 min of Mch stimulation and compared between the two conditions (right panel). ***p<0.001 with extracellular Ca2+ (n = 9 cells) versus without the Ca2+ (n = 12 cells); Student's unpaired t-test. (C) Ca2+ stores remained functional in the absence of extracellular Ca2+. The cells were placed in the absence of extracellular Ca2+ for ∼15 min, and then stimulated with two 10 µM Mch pulses 15 min apart. The chart on the right indicates that two Mch administrations produced comparable Ca2+ responses, i.e., Ca2+ stores are intact under experimental conditions in the present study. NS, p>0.05 for the response in the first pulse of Mch versus that in the second pulse, Student's paired t-test, n = 10. ΔF/F0 for (B) and (C) are the average over the entire cell. (TIFF) [file pbio.1001501.s003.tiff]

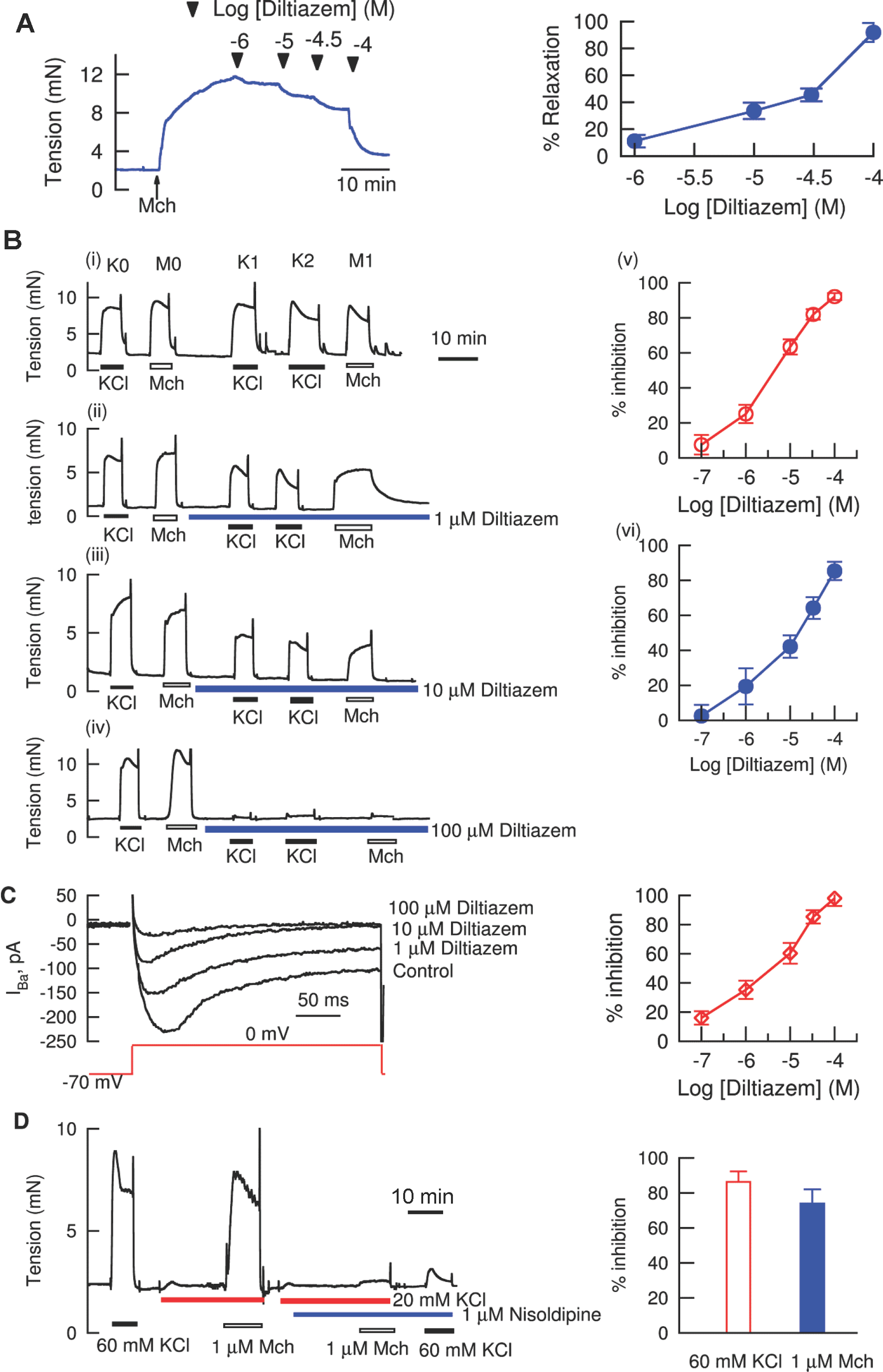

Supplement: Figure S4 — Role of L-type Ca2+ channel activation in Mch-induced contraction in mouse airway. (A) Left panel: L-type VDCC blocker diltiazem dose-dependently reversed Mch-induced contraction (using tension as a proxy measure). Right panel: results for n = 6 airway rings. % relaxation was calculated the same as in Figure S2B. (B) Diltiazem inhibited Mch- and KCl-induced contraction in a dose-dependent manner. (i) Once equilibrated, airway rings generated stable responses to 60 mM KCl (i.e., K0, K1, and K2), and to 1 µM Mch (i.e., M0 and M1) over a time span longer than 1 hr. (ii, iii, iv) show representative responses to KCl and Mch in the presence of diltiazem at 1 µM, 10 µM, and 100 µM, respectively. Two pulses of KCl (K1 and K2) were administrated before Mch (M1) to facilitate diltiazem inactivation of L-type Ca2+ channels. (v) Dose-response curve for diltiazem-mediated inhibition of contraction by KCl. Data are mean ± SEM (n = 5); % inhibition = (force by K0 − force by K2)/(force by K0)×100. (vi) Dose-response curve for diltiazem-mediated inhibition of contraction by Mch. Data are mean ± SEM (n = 5); % inhibition = (force by M0 – force by M1)/(force by M0)×100. Forces are measured at their peak, excluding the noise spikes due to the washing out of KCl or Mch. (C) Diltiazem inhibited L-type VDCC currents in a dose-dependent manner. Diltiazem was added to the bath solution cumulatively. Once at equilibrium at each level of diltiazem, cells were stimulated with a train of ten voltage pulses from −70 mV to 0 mV (inset) at 10 s intervals. Left panel displays patch clamp recordings of L-type Ca2+ currents in the control and in the presence of diltiazem at the given concentration (each in response to the tenth voltage pulse), and the right panel depicts the effect of diltiazem on the peak current at different concentrations. Ba2+ was used as the charge carrier. Data are mean ± SEM (n = 6); % inhibition = (peak current of the control − peak current at given diltiazem concentration)/ [file pbio.1001501.s004.tiff]

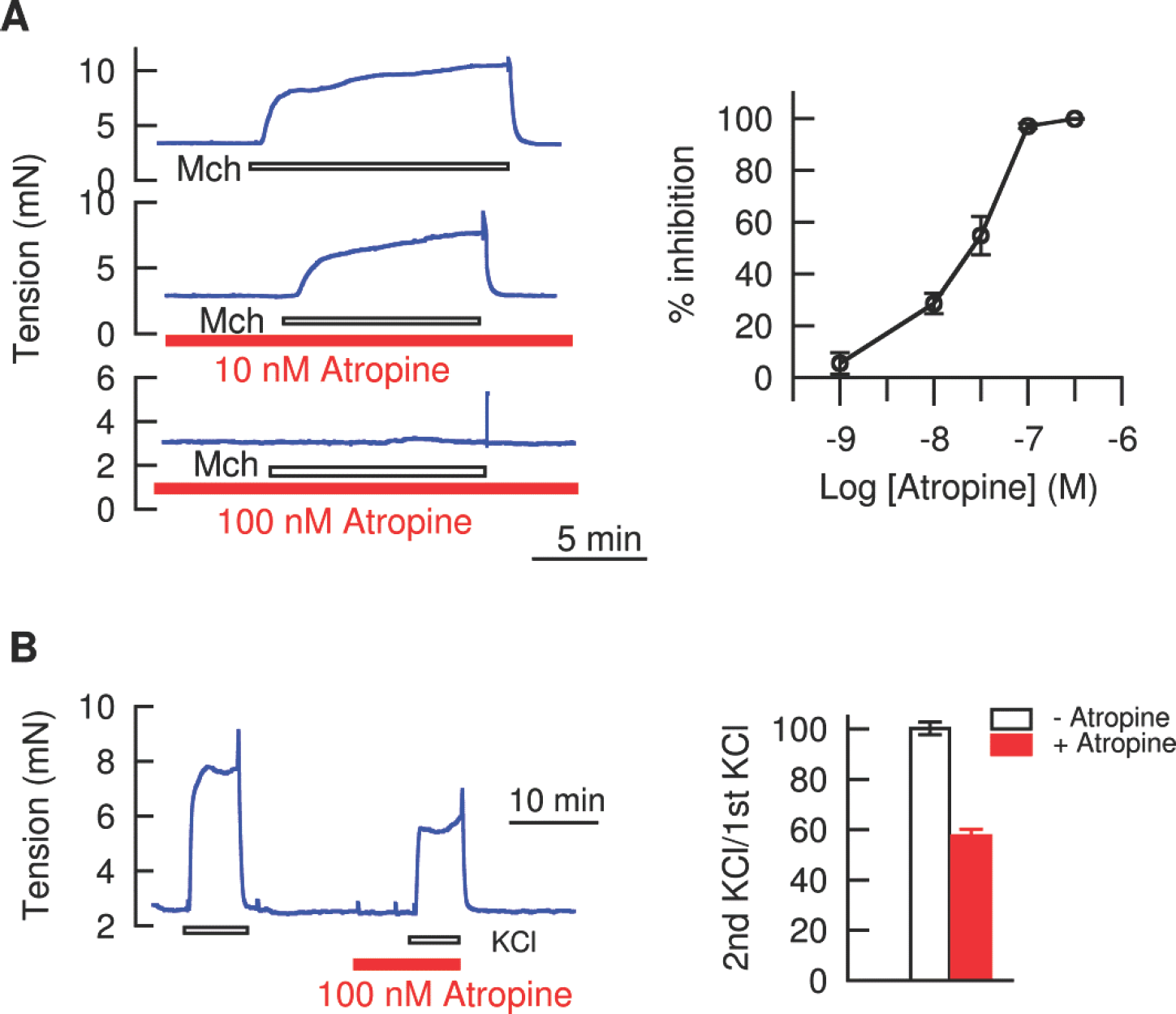

Supplement: Figure S5 — KCl activates VDCCs in cholinergic nerves and ASM. (A) Atropine dose-dependently inhibited Mch-induced mouse airway contraction. The left panels display representative contractile responses to 3 µM Mch in the absence or the presence of atropine as marked near the traces, and the right panel shows the mean values (mean ± SEM; n = 4–8) of inhibition by atropine of contraction evoked by 3 µM Mch. % inhibition = (maximal force from the control − maximal force with atropine)/maximal force from the control ×100. (B) Atropine inhibited KCl-induced mouse airway contraction. The left panel shows a representative tension recording in response to 60 mM KCl before and after 100 nM atropine, as marked beneath. The right panel shows the summarized results as the ratio of the force generated by the second KCl pulse over first KCl pulse (mean ± SEM; n = 6 for the time matched controls, n = 28 for atropine). (TIFF) [file pbio.1001501.s005.tiff]

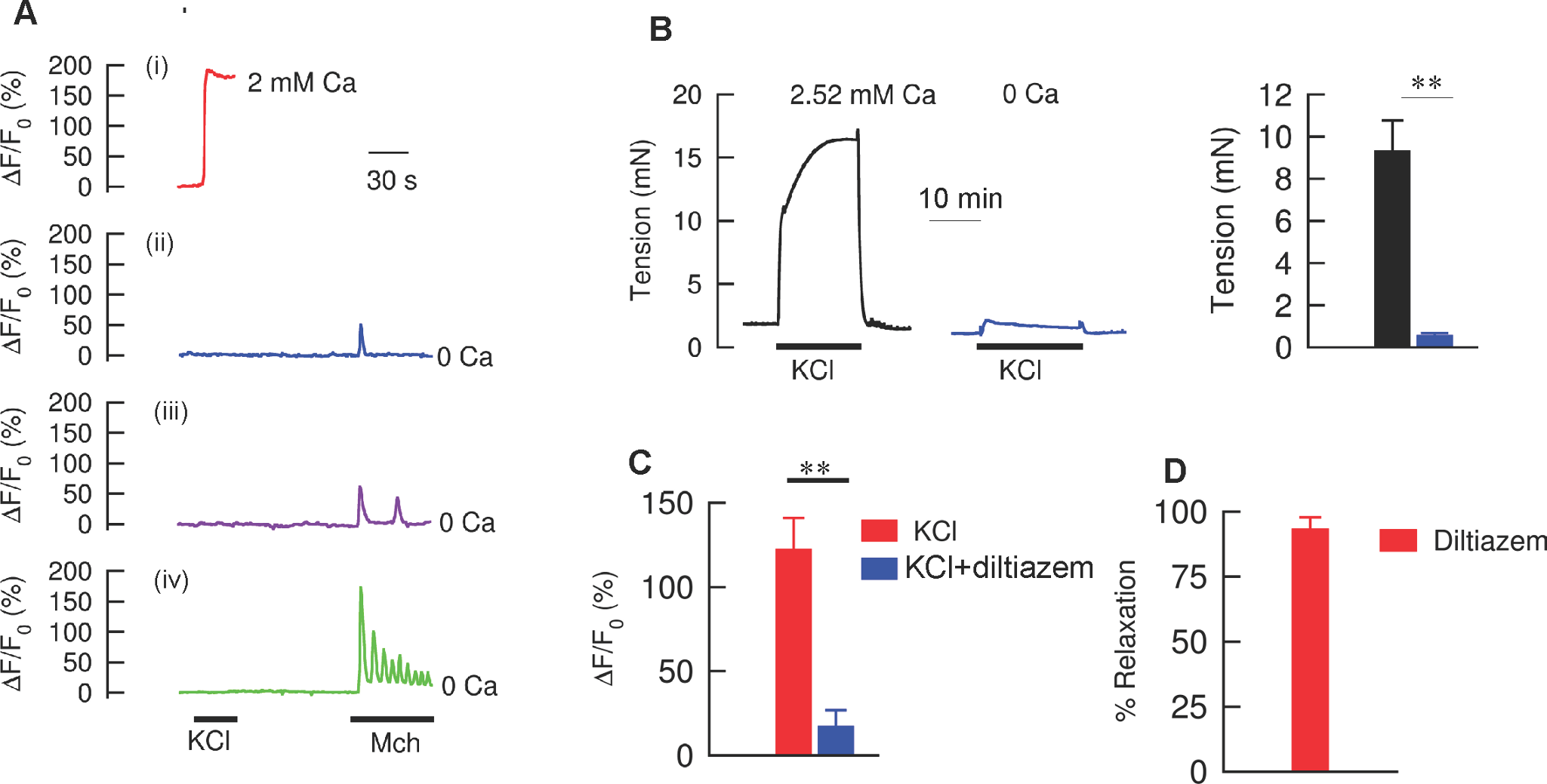

Supplement: Figure S6 — KCl activates L-type VDCCs to increase [Ca2+]i and cause contraction in mouse ASM. (A) KCl failed to generate any global [Ca2+]i increase in the absence of extracellular Ca2+ in isolated single ASM cells. (i) A representative [Ca2+]i response to 60 mM KCl in the presence of extracellular Ca2+. (ii, iii, iv) three examples showing that the same concentration of KCl did not increase Ca2+ in the zero Ca2+ medium. This failure was not due to the depletion of intracellular Ca2+ stores because 10 µM Mch still induced Ca2+ release either as a single peak or as oscillations. Eight cells gave rise to similar responses. ΔF/F0 is the average over the entire cell. (B) KCl (60 mM) caused virtually no increase in tension in the absence of extracellular Ca2+. The airways were placed in the Ca2+ free solution for 15 min before the measurement commenced. Left panel shows a pair of representative recordings and right panel the average results. **p<0.01, Student's paired t-test, n = 6 independent experiments. (C) KCl (60 mM)-induced increase in [Ca2+]i was markedly inhibited by prior application of L-type VDCC blocker diltiazem (100 µM). **p<0.01, Student's paired t-test, n = 9 for each conditions. (D) Diltiazem (100 µM) relaxed 60 mM KCl-induced contraction of mouse airways. Data are mean ± SEM (n = 6 independent experiments), and % relaxation definition and analysis are the same as in Figure S2C. (TIFF) [file pbio.1001501.s006.tiff]
